# Improving accessibility to radiotherapy services in Cali, Colombia: cross-sectional equity analyses using open data and big data travel times from 2020

**DOI:** 10.1186/s12939-024-02211-6

**Published:** 2024-08-15

**Authors:** Luis Gabriel Cuervo, Carmen Juliana Villamizar, Daniel Cuervo, Pablo Zapata, Maria B. Ospina, Sara Marcela Valencia, Alfredo Polo, Ángela Suárez, Maria O. Bula, J. Jaime Miranda, Gynna Millan, Diana Elizabeth Cuervo, Nancy J. Owens, Felipe Piquero, Janet Hatcher-Roberts, Gabriel Dario Paredes, María Fernanda Navarro, Ingrid Liliana Minotta, Carmen Palta, Eliana Martínez-Herrera, Ciro Jaramillo, on behalf of the AMORE Project Collaboration, Freddy Enrique Agredo Lemos, Juan Camilo Arboleda, German Avila Rodriguez, Alberto Concha-Eastman, Ingrid Faber, Oscar H. Franco, Crhistian Camilo Garcia Altamirano, Rodrigo Guerrero Velasco, Déborah Itriago, Edith Alejandra Martin, Fernando Rafael Martinez Arambula, Maria Fernanda Merino Juarez, Jackeline Murillo-Hoyos, Ana Ortigoza, Lyda Osorio, David Paredes-Zapata, Luis Fernando Pinilla, Oscar Rojas Rentería, Myriam Rosero Hernández, María Fernanda Tobar-Blandón

**Affiliations:** 1 Departamento de Pediatría, de Obstetricia y Ginecología y de Medicina Preventiva y Salud Pública. Facultad de Medicina - Edificio M, Universitat Autònoma de Barcelona, Campus Universitario UAB, 08193 Bellaterra, (Cerdanyola del Vallès) Cataluña Spain; 2 Academia Nacional de Medicina de Colombia, Cra. 7ª # 69-11, 110231 Bogotá, Colombia; 3 Johns Hopkins Bloomberg School of Public Health, Wolfe Street Building, W1015, Baltimore, MD 21205 USA; 4 IQuartil SAS, Cra 13A # 107A-47, 110111 Bogotá, Colombia; 5 Department of Public Health Sciences, Faculty of Health Sciences, Queen’s University, Carruthers Hall 204, Kingston, ON K7L 3N6 Canada; 6 Universidad Nacional de Colombia, Ave Cra. 30 # 45-03, 111321 Bogotá, Colombia; 7 Facultad de Medicina, Universidad de Antioquia, Cra. 51D # 62-29, Medellín, Antioquia 050010 Colombia; 8 Technical Cooperation and Capacity Development, City Cancer Challenge Foundation, 9 Rue du Commerce, Geneva, 1204 Switzerland; 9 Independent Researcher, 110221 Bogotá, Colombia; 10 CRONICAS Center of Excellence in Chronic Diseases, Universidad Peruana Cayetano Heredia, Av. Armendáriz 445 - Miraflores, 15074 Lima, Peru; 11 Sydney School of Public Health, Faculty of Medicine and Health, University of Sydney, Camperdown, NSW 2006 Australia; 12 Universidad del Valle, Cali, Valle del Cauca 760032 Colombia; 13 Junta Nacional de Calificación de Invalidez [National Disability Board of Colombia], 110111 Bogotá, Colombia; 14 Independent Content and Communications Consultant, Fairfax, VA 22032 USA; 15 Patient Advocate and Author of an Autopathography, 110231 Bogotá, Colombia; 16 WHO Collaborating Centre for Knowledge Translation and Health Technology Assessment for Health Equity, Bruyère Research Institute, University of Ottawa, Ottawa, ON K1N 5C8 Canada; 17 Independent Consultant On Emergency Medicine and Humanitarian Response, 110111 Bogotá, Colombia; 18 Regional Director, City Cancer Challenge Foundation, 110111 Bogotá, Colombia; 19 ProPacífico, Calle 35 Norte #6A Bis - 100, 760046 Cali, Valle del Cauca Colombia; 20 National Faculty of Public Health, Universidad de Antioquia, Cl. 62 #52-59, La Candelaria, 050010 Medellín, Antioquia Colombia; 21 JHU-UPF Public Policy Center, Departament de Ciències Polítiques I Socials, Universitat Pompeu Fabra (UPF), Barcelona School of Management (UPF-BSM), Barcelona, Cataluña Spain; 22 School of Civil and Geomatic Engineering of the Universidad del Valle, Cali, Valle del Cauca 760032 Colombia

## Abstract

In this study, we evaluated and forecasted the cumulative opportunities for residents to access radiotherapy services in Cali, Colombia, while accounting for traffic congestion, using a new people-centred methodology with an equity focus. Furthermore, we identified 1–2 optimal locations where new services would maximise accessibility. We utilised open data and publicly available big data. Cali is one of South America's cities most impacted by traffic congestion.

**Methodology:** Using a people-centred approach, we tested a web-based digital platform developed through an iterative participatory design. The platform integrates open data, including the location of radiotherapy services, the disaggregated sociodemographic microdata for the population and places of residence, and big data for travel times from Google Distance Matrix API. We used genetic algorithms to identify optimal locations for new services. We predicted accessibility cumulative opportunities (ACO) for traffic ranging from peak congestion to free-flow conditions with hourly assessments for 6–12 July 2020 and 23–29 November 2020. The interactive digital platform is openly available.

**Primary and secondary outcomes:** We present descriptive statistics and population distribution heatmaps based on 20-min accessibility cumulative opportunities (ACO) isochrones for car journeys. There is no set national or international standard for these travel time thresholds. Most key informants found the 20-min threshold reasonable. These isochrones connect the population-weighted centroid of the traffic analysis zone at the place of residence to the corresponding zone of the radiotherapy service with the shortest travel time under varying traffic conditions ranging from free-flow to peak-traffic congestion levels. Additionally, we conducted a time-series bivariate analysis to assess geographical accessibility based on economic stratum. We identify 1–2 optimal locations where new services would maximize the 20-min ACO during peak-traffic congestion.

**Results:** Traffic congestion significantly diminished accessibility to radiotherapy services, particularly affecting vulnerable populations. For instance, urban 20-min ACO by car dropped from 91% of Cali’s urban population within a 20-min journey to the service during free-flow traffic to 31% during peak traffic for the week of 6–12 July 2020. Percentages represent the population within a 20-min journey by car from their residence to a radiotherapy service. Specific ethnic groups, individuals with lower educational attainment, and residents on the outskirts of Cali experienced disproportionate effects, with accessibility decreasing to 11% during peak traffic compared to 81% during free-flow traffic for low-income households. We predict that strategically adding sufficient services in 1–2 locations in eastern Cali would notably enhance accessibility and reduce inequities. The recommended locations for new services remained consistent in both of our measurements.

These findings underscore the significance of prioritising equity and comprehensive care in healthcare accessibility. They also offer a practical approach to optimising service locations to mitigate disparities. Expanding this approach to encompass other transportation modes, services, and cities, or updating measurements, is feasible and affordable. The new approach and data are particularly relevant for planning authorities and urban development actors.

Video Abstract

**Supplementary Information:**

The online version contains supplementary material available at 10.1186/s12939-024-02211-6.

## Español

En este estudio, evaluamos y pronosticamos las oportunidades acumulativas para que los residentes accedan a los servicios de radioterapia en Cali, Colombia, teniendo en cuenta la congestión del tráfico, utilizando una nueva metodología centrada en las personas con un enfoque de equidad. Además, identificamos 1-2 ubicaciones óptimas donde los nuevos servicios maximizarían la accesibilidad. Utilizamos datos abiertos y macrodatos disponibles públicamente. Cali está entre las ciudades Sudamericanas más afectadas por la congestión del tráfico.

**Metodología:** Usando un enfoque centrado en las personas, probamos una plataforma digital basada en la web desarrollada a través de un diseño participativo iterativo. La plataforma integra datos abiertos, incluyendo la ubicación de los servicios de radioterapia, los microdatos sociodemográficos desagregados de la población y los lugares de residencia, y los macrodatos de tiempos de viaje de la API de Google Distance Matrix. Usamos algoritmos genéticos para identificar ubicaciones óptimas para nuevos servicios. Pronosticamos oportunidades acumulativas de accesibilidad (ACO, por sus siglas en inglés) para el tráfico que va desde la congestión máxima hasta condiciones de flujo libre, con evaluaciones horarias hechas del 6 al 12 de julio de 2020 y del 23 al 29 de noviembre de 2020. La plataforma digital interactiva está públicamente disponible.

**Resultados Primarios y Secundarios:** Presentamos estadísticas descriptivas y mapas de calor de la distribución de la población basados en isócronas de ACO de 20 minutos para viajes en coche. No existe un estándar nacional o internacional establecido para estos umbrales de tiempo de viaje. La mayoría de los informantes clave encontraron razonable el umbral de 20 minutos. Estas isócronas conectan el centroide poblacional ponderado de la zona de análisis de tráfico del lugar de residencia con la zona correspondiente del servicio de radioterapia con menor tiempo de viaje bajo condiciones variables de tráfico, que van desde el flujo libre hasta niveles de congestión de tráfico máximo. Además, realizamos un análisis bivariado de series temporales para evaluar la accesibilidad geográfica basada en el estrato económico. Identificamos 1-2 ubicaciones óptimas donde los nuevos servicios maximizarían el ACO de 20 minutos durante la congestión máxima del tráfico.

**Resultados:** La congestión del tráfico redujo significativamente la accesibilidad a los servicios de radioterapia, afectando particularmente a las poblaciones vulnerables. Por ejemplo, el ACO urbano de 20 minutos en coche se redujo del 91% de la población urbana de Cali para viajes de hasta 20 minutos al servicio con flujo libre de tráfico, al 31% cuando hay congestión pico de tráfico durante la semana del 6 al 12 de julio de 2020. Los porcentajes representan la población con viajes de hasta 20 minutos en coche desde la residencia hasta el servicio de radioterapia. Grupos étnicos específicos, individuos con menor nivel educativo y residentes en las afueras de Cali experimentaron efectos desproporcionados, con la accesibilidad disminuyendo al 11% durante el tráfico máximo en comparación con el 81% durante el tráfico de flujo libre para hogares de bajos ingresos. Predecimos que agregar estratégicamente suficientes servicios en 1-2 ubicaciones en el este de Cali mejoraría notablemente la accesibilidad y reduciría las inequidades. Las ubicaciones recomendadas para los nuevos servicios se mantuvieron consistentes en nuestras dos mediciones.

Estos hallazgos subrayan la importancia de priorizar la equidad y la atención integral en la accesibilidad a la atención médica. También ofrecen un enfoque práctico para optimizar las ubicaciones de los servicios para mitigar las disparidades. Es factible y accesible expandir este enfoque para abarcar otros modos de transporte, servicios y ciudades, o actualizar las mediciones. El nuevo enfoque y los datos son particularmente relevantes para las autoridades de planificación y los actores del desarrollo urbano.

## Português

Neste estudo, avaliamos e previmos as oportunidades cumulativas para os residentes acessarem serviços de radioterapia em Cali, Colômbia, levando em consideração o congestionamento do tráfego, utilizando uma nova metodologia centrada nas pessoas com um foco na equidade. Além disso, identificamos 1-2 locais ideais onde os novos serviços poderiam maximizar a acessibilidade. Utilizamos dados abertos e big data disponíveis publicamente. Cali está entre as cidades sulamericanas mais afetadas pela congestionamento do tráfego.

**Metodologia:** Usando uma abordagem centrada em pessoas, testamos uma plataforma digital baseada na web que foi desenvolvida através de um design participativo iterativo. A plataforma integra dados abertos, incluindo a localização dos serviços de radioterapia, microdados sociodemográficos desagregados por população e locais de residência, e big data da API Google Distance Matrix para os tempos de viagem. Utilizamos algoritmos genéticos para identificar locais ideais para novos serviços. Previmos oportunidades cumulativas de acessibilidade (ACO, por suas siglas em inglês) para o tráfego que varia desde o congestionamento máximo até condições de fluxo livre, com avaliações horárias de 6 a 12 de julho de 2020 e de 23 a 29 de novembro de 2020. A plataforma digital interativa está disponível publicamente.

**Resultados Primários e Secundários:** Apresentamos estatísticas descritivas e mapas de calor da distribuição populacional baseados em isócronas de ACO de 20 minutos para viagens de carro. Não existe um padrão nacional ou internacional estabelecido para esses limites de tempo de viagem. A maioria dos entrevistados-chave considerou razoável o limite de 20 minutos. Essas isócronas conectam o centroide ponderado pela população da zona de análise de tráfego no local de residência e sua zona correspondente do serviço de radioterapia com o menor tempo de viagem sob condições variáveis de tráfego (que vão desde fluxo livre até níveis máximos de congestionamento do tráfego). Além disso, realizamos uma análise bivariada de séries temporais para avaliar a acessibilidade geográfica baseada na estratificação econômica. Identificamos 1-2 locais ideais onde os novos serviços maximizariam a ACO de 20 minutos durante o pico de congestionamento do tráfego.

**Resultados:** O congestionamento do tráfego reduziu significativamente a acessibilidade aos serviços de radioterapia, afetando particularmente as populações vulneráveis. Por exemplo, a ACO urbana de 20 minutos de carro foi reduzida de 91% durante um fluxo livre de tráfego, para 31% durante picos de congestionamento de tráfego entre a semana de 6 a 12 de julho de 2020. As porcentagens representam a população com viagens de até 20 minutos de carro de sua residência até o serviço de radioterapia. Grupos étnicos específicos, indivíduos com menor nível educacional e residentes nos arredores de Cali experimentaram resultados desproporcionais, com a acessibilidade diminuindo para 11% durante o tráfego com congestionamento máximo em comparação com 81% durante o fluxo livre de tráfego para domicílios de baixa renda. Prevemos que adicionar estrategicamente serviços suficientes em 1-2 locais no leste de Cali melhoraria significativamente a acessibilidade e reduziria as desigualdades. Os locais recomendados para os novos serviços permaneceram consistentes em ambas medições. Esses resultados ressaltam a importância de priorizar a equidade e o atendimento integral na acessibilidade aos cuidados de saúde. Eles também oferecem uma abordagem prática para otimizar os locais dos serviços a fim de minimizar as desigualdades. A expansão dessa abordagem para abranger outros modos de transporte, serviços e cidades, ou a atualização das medições, é viável e acessível. A nova abordagem e os dados são particularmente relevantes para as autoridades de planejamento e os agentes do desenvolvimento urbano.

## Français

Dans cette étude, nous avons évalué et prévu les opportunités cumulées pour les résidents d'accéder aux services de radiothérapie à Cali, en Colombie, tout en tenant compte de la congestion du trafic en utilisant une nouvelle méthodologie centrée sur les personnes avec axée sur l'équité. De plus, nous avons identifié 1 à 2 emplacements optimaux où de nouveaux services maximiseraient l'accessibilité. Nous avons utilisé des données ouvertes et des macrodonnées tenues à disposition du public. Cali est l'une des villes d'Amérique du Sud les plus touchées par la congestion du trafic.

**Méthodologie:** En utilisant une approche centrée sur les personnes, nous avons testé une plateforme numérique basée sur le web développée à travers une conception participative itérative. La plateforme intègre des données ouvertes, y compris la localisation des services de radiothérapie, les microdonnées sociodémographiques désagrégées de la population et les lieux de résidence, ainsi que les mégadonnées des temps de trajet de l'API Google Distance Matrix. Nous avons utilisé des algorithmes génétiques pour identifier les emplacements optimaux pour de nouveaux services. Nous avons prévu les opportunités cumulatives d'accessibilité (ACO, désignant l'acronyme en anglais) pour le trafic allant de la congestion maximale aux conditions de flux libre, avec des évaluations horaires du 6 au 12 juillet 2020 et du 23 au 29 novembre 2020. La plateforme numérique interactive est disponible publiquement.

**Résultats Primaires et Secondaires:** Nous présentons des statistiques descriptives et des cartes thermiques de la distribution de la population basées sur des ACO de 20 minutes pour les trajets en voiture. Il n'existe pas de norme nationale ou internationale établie pour ces seuils de temps de trajet. La majorité des informateurs clés ont trouvé le seuil de 20 minutes raisonnable. Ces isochrones relient le centroïde pondéré par la population de la zone d'analyse du trafic au lieu de résidence à la zone correspondante du service de radiothérapie avec le temps de trajet le plus court sous des conditions de trafic variables allant du flux libre aux niveaux de congestion de trafic maximum. De plus, nous avons réalisé une analyse bivariée des séries chronologiques pour évaluer l'accessibilité géographique en fonction de la stratification économique. Nous avons identifié 1 à 2 emplacements optimaux où de nouveaux services maximiseraient l'ACO de 20 minutes pendant la congestion maximale du trafic.

**Résultats:** La congestion du trafic a considérablement réduit l'accessibilité aux services de radiothérapie, affectant particulièrement les populations vulnérables. Par exemple, l'ACO urbain de 20 minutes en voiture est passé de 91 % de la population urbaine de Cali pour des trajets de jusqu'à 20 minutes vers le service avec un flux de trafic libre à 31 % lors des pics de congestion de trafic pendant la semaine du 6 au 12 juillet 2020. Les pourcentages représentent la population avec des trajets de jusqu'à 20 minutes en voiture depuis la résidence jusqu'au service de radiothérapie. Des groupes ethniques spécifiques, des individus ayant un niveau d'éducation inférieur et des résidents des périphéries de Cali ont subi des effets disproportionnés, avec une accessibilité diminuant à 11 % pendant le trafic maximal par rapport à 81 % pendant le flux de trafic libre pour les ménages à faible revenu. Ajouter suffisamment de services à 1-2 emplacements stratégiques dans l'est de Cali a le potential d’améliorer considérablement l'accessibilité et réduirait les inégalités. Les emplacements recommandés pour les nouveaux services sont restés cohérents dans nos deux mesures. Ces conclusions soulignent l'importance de prioriser l'équité et une prise en charge globale dans le cadre de l'accessibilité aux soins de santé. Elles offrent également une approche pratique pour optimiser les emplacements des services afin de réduire les disparités. Il est faisable et abordable d'étendre cette approche pour inclure d'autres modes de transport, services et villes, ou pour mettre à jour les mesures. La nouvelle approche et les données sont particulièrement pertinentes pour les autorités de planification et les acteurs du développement urbain.

## What we know on this topic

The benefits of setting up new hospitals and health services in areas that are accessible and convenient to patients are well known, particularly for vulnerable populations. Moreover, people increasingly prefer to visit healthcare sites within a short trip where they spend their daily lives rather than embarking on long journeys or sitting in traffic. Direct and indirect costs associated with long journeys impact adherence, clinical outcomes, and patients' and their family's quality of life and economy. Having conveniently located health services contributes to spatial justice and health equity.

This report expands on our studies for haemodialysis and tertiary care emergency services. Those studies assessed the feasibility of generating dynamic geographical accessibility measurements by integrating open data and big data in participatory processes with stakeholders while incorporating an equity perspective. Our previous findings also identified eastern Cali as the priority area where new services would optimise accessibility. However, the recommended sectors varied as the distribution of haemodialysis and tertiary care emergency services differs from radiotherapy services.

## What this study adds

This study introduces an innovative methodology and tool codesigned with stakeholders and data end users to be applied by the public and private sectors to strategically plan the establishment of new healthcare sites, focusing on urban accessibility to critical services such as radiotherapy. In addition to the participatory approach, the tool relies on relatable cartography and descriptive statistics to effectively communicate assessments and predictions regarding urban accessibility to radiotherapy services in Cali. It identifies optimal locations for new radiotherapy services, considering factors such as traffic congestion, and offers crucial data-driven insights for enhanced health services planning. This study tests a practical approach involving relevant stakeholders in response to numerous calls from leaders and intersectoral organisations advocating for participatory intersectoral actions to align the urban and health agendas by focusing on equity and social justice.

## How might this study affect research, practice, or policy?

This study supports a fundamental shift in how accessibility measurements for health services are approached when traffic congestion is a factor. This approach equips advocates and stakeholders to participate in urban and health services planning with accurate and relatable data, fostering intersectoral and multistakeholder collaborations. The study also offers an interactive public platform for testing data and scenarios.

Governments, local authorities, and health/insurance companies benefit from incorporating these measurements into their health and urban planning policies. Civic organisations, government, and patient groups can explore how to monitor and link traffic congestion with equity and accessibility. This integration can improve overall accessibility, efficiency, and public participation. Moreover, the study can influence policies prioritising public health by underscoring the critical role of addressing traffic congestion in ensuring equitable access to health services. It sheds light on the impact of travel times as barriers to adherence and access to health services, particularly in conjunction with appointment opportunities, authorisations, and the fair allocation of subsidies.

## Background

Traffic congestion reduces accessibility to health services and might affect population groups differently. However, measuring congestion has been elusive for practical reasons [1–3]. Most measurements have focused on average travel times or distances, which is problematic since fixed average estimates are misleading when traffic fluctuates and varies across sectors [3–7]. In these situations, residents often plan their movements based on travel times rather than distance due to the rarity of smooth, free-flow traffic that usually allows for predictable and economical transportation. In this article, we will refer to geographical accessibility measurements that consider temporospatial variations in traffic congestion as dynamic geographical accessibility measurements (DGAMs).

For example, a 2017 study using fixed estimates to measure accessibility to tertiary care emergency services in Cali, Colombia, yielded results like free-flow traffic in the first hours of weekdays, which a 2020 DGAM also captured. However, Cali is among the most traffic-congested cities in South America [8–10]. The DGAM showed that under usual traffic congestion conditions, 15-minute urban accessibility to tertiary care emergency services by car dropped from 84% to 38%, revealing a previously overlooked problem [11–15]. Reduced accessibility can adversely impact the overall quality of healthcare, life, and health outcomes.

DGAM studies are increasingly becoming available for health services [2, 6, 7, 16–19]. However, studies on accessibility to service provision are often done with minimal stakeholder involvement and disregard other knowledge translation recommendations [1, 20–27]. Furthermore, these studies employ complex methods and communicate findings to a specialised audience, which may not always be directly related to healthcare. While such assessments are very much needed in public health, for example, to understand the interactions between patients and services, consider service agglomeration and spatial accessibility, or geographical distribution of service users and providers, [28, 29] reports are frequently of limited practical value in multistakeholder or intersectoral deliberations; they are usually cumbersome to update and rarely offer interactive options, rendering them ineffective for testing different scenarios, assumptions, monitoring, and planning [3, 13, 28, 30–34]. The AMORE Project and the OnTIME consortium are addressing some of these limitations, and this report for radiotherapy services adds to these. This report describes an innovative integrative approach that strategically engages diverse stakeholders involved in providing and receiving cancer treatment [5, 12, 18, 35–37]. While this report focuses on radiotherapy, the broader study tests a methodological approach applicable to diverse services within and beyond the health sector, providing an equity perspective and elements that differentiate it from existing approaches. For example, it points to where to place new services, going beyond describing service agglomeration to consider traffic congestion and urban sprawl, as well as the city’s mobility network with the control measures that sometimes exacerbate inequities [36, 38].

A research project in Cali’s informal settlements of the western peripheral Commune 18 produced qualitative data highlighting the problems people face when commuting to ambulatory cancer and dialysis services they require several times weekly [39–41]. These patients and their families can face financial hardship because of the high costs, direct and indirect (e.g., lost wages, missed opportunities) [42–49] (Supplementary Material 1).

Patients in Cali’s slopes and other peripheral areas offer striking testimonies of the impact of their commute, frequently struggling to reach a paved road where a taxi can pick them up. Those in a wheelchair face the additional hurdle of finding a taxi that carries the chair. They sometimes struggle to find the money to pay for transportation, risking their well-being [39–41] (Supplementary Material 1).

Radiotherapy is an essential component of cancer care [50]. Radiotherapy capacites are strained and strategic efficient planning is needed to maximise efficiencies [51–53]. Comprehensive quality cancer treatments involving radiation therapy encompass additional interventions to improve physical, social, and mental well-being, providing support, education, and rehabilitation for patients and carers [54–57]. Radiotherapy is typically administered on an outpatient basis, requiring patients to visit the radiotherapy facility daily throughout the treatment course, which may span several weeks. This fact adds complexity to the analysis of accessibility to service provision.

Data and insights are needed to understand the factors contributing to poor spatial (geographical) accessibility to radiotherapy services and to address health equity and social justice [2, 58–61]. Measuring the effects of traffic congestion on accessibility among populations enables data-driven approaches to address underlying issues. These include land-use policies or other factors contributing to market failure and service scarcity in underserved areas, including revenue, availability of specialists, or insecurity. Individual ability to pay is not directly linked to service payments in Colombia because health insurance schemes make most payments but need to cover transportation systematically [62]. A systematic review assessing the use of geographical information systems for radiotherapy planning found no studies providing an equity perspective that considered traffic congestion in low or middle-income countries, part of a multistakeholder coalition, or following a participatory people-centred approach [63]. We found no other studies co-designed with end users and stakeholders providing dynamic spatiotemporal accessibility measurements and predictions for health services [1]. Participation of beneficiaries and health services users in health services research promotes trust, effectiveness, community rights and several other benefits [37, 64].

Transportation subsidies are increasingly available in Colombia to treat high-cost diseases like cancer. However, these subsidies can perpetuate inequities because they need to account for factors that define out-of-pocket and indirect costs, such as travel time or distance. Furthermore, implementing these subsidies in 2020 was not systematic, leaving disadvantaged populations facing accessibility challenges [42, 65–68]. Poor geographical accessibility to cancer treatments diminishes the quality of care and is associated with poor clinical outcomes [56, 63, 69–73].

In this study, we aim to assess the impact of traffic congestion on the accessibility of radiotherapy services within urban Cali; explore the connections between sociodemographic factors influencing health equity, traffic congestion patterns, and the accessibility of healthcare services; and predict the enhanced accessibility of radiotherapy services by strategically introducing facilities in 1–2 key locations. This predictive model is designed to optimise service accessibility, cater to the healthcare needs of Cali’s diverse population, and be accessible to diverse stakeholders [13, 29, 74].

## Methods

This study outlines a new strategic people and equity-centred approach developed in 2020 to generate DGAMs for health services, combining a range of methods, novel processes, and ideas. The investigators comprise a multistakeholder international collaboration with governmental and non-governmental contributors taking a whole-of-system approach on health services accessibility seeking to promote data-driven health services planning and health equity [75, 76]. The project examined tertiary care emergency services, for which time is critical, and two outpatient services that require frequent and prolonged attendance: haemodialysis and radiotherapy. This report focuses on the latter [38].

We developed the AMORE web-based platform to integrate open sources such as the adjusted national census microdata, matching it to a Traffic Analysis Zone (TAZ), and travel times between TAZ population-weighed centroids. This allowed us to obtain the dynamic spatial-temporal accessibility measurements (DSTAM) [12, 36, 38, 77]. The platform followed a participatory design approach with engagement from data scientists, researchers, collaborators and stakeholders throughout the design, implementation and reporting [13, 25, 38]. We held twenty-eight interviews and discussion groups with key informants, experts and stakeholders to get feedback and insights as the AMORE Platform was developed, leading to enhanced prototypes and presenting data in ways relatable to end users [13, 25, 38, 75]. Interviews with end users were expanded in 2023 as part of an innovation challenge and an assessment [13, 78]. We identified radiotherapy services in Cali using the Colombian National Health Services Registry (Supplementary Material 5). We obtained big data on travel times from Google Distance Matrix API for the weeks of July 6–12 and 23–29 November 2020 to perform situational analyses. On July 3, 2020, we downloaded predicted travel times for the week of 6–12 July 2021. For the week of 23–29 November 2020, we downloaded the expected times on 27 October 2020. None of these weeks had holidays, and they offered an opportunity to explore variations during the COVID-19 pandemic [12, 36, 38].

The AMORE web-based platform displays accessibility cumulative opportunities (ACO), a contour measure also known as isochronic indices, a metric for the number or proportion of opportunities of reaching a destination (i.e., radiotherapy service) within a specified travel time (i.e., 20 minutes by car) [9, 79]. It measured travel times from the population-weighed centroid of each residential TAZ to the centroid of the TAZ with the shortest travel time [38]. We used advanced heuristic genetic algorithms to predict the 1–2 locations where new services would maximise the accessibility across Cali. Those algorithms factored in disaggregated accessibility data by the sociodemographic population characteristics [36].

The 20-minute threshold was arbitrary and found reasonable by focus groups discussing commuting times for radiotherapy or haemodialysis. There is no set national or international standard for these travel time thresholds [38]. This was complemented by grading accessibility by socioeconomic stratum of housing at 10-minute intervals to provide deeper perspectives into social justice and health equity [12, 36, 38, 61].

### Study population and setting

**Fig. 1:**
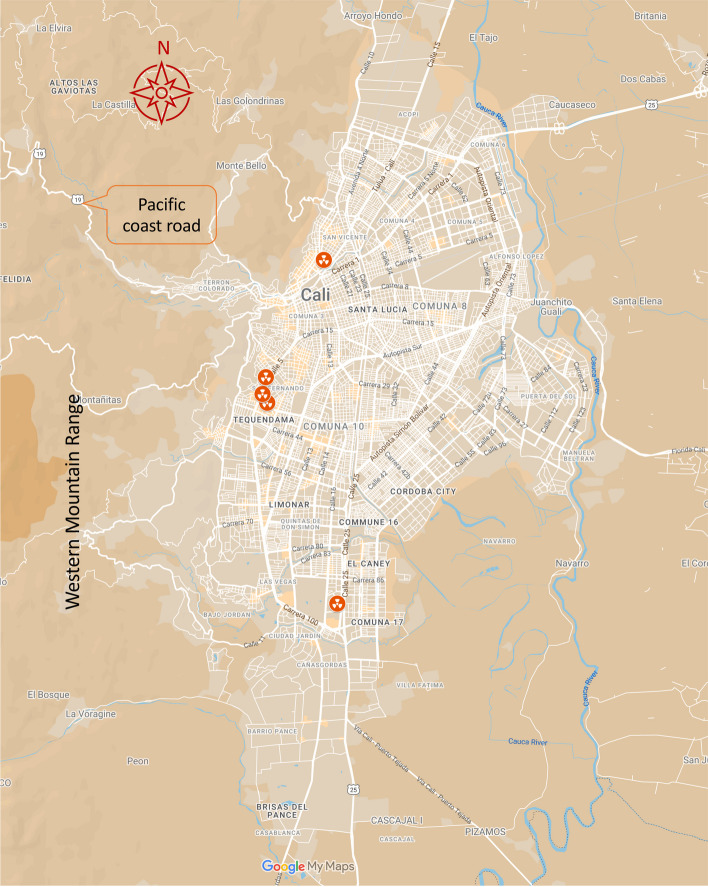
Location of Cali’s radiotherapy services in 2020, with compass rose, mountain range, and road indications for clarity [80]

Cali, a tropical city nested in a valley, lies between the western Andean mountains and the west bank of the Cauca River. Some of its neighbourhoods cover the western Andean slopes, including those along the road leading to the Pacific in northwest Cali on its westernmost border. Cali is the only major Colombian city with access to the Pacific c﻿oast (Fig. 1). This study focused on the urban population of Cali, as estimated for 2020 after updating the recorded 2018 national census microdata, as described in the research protocol.

Cali consisted of 22 communes in 2020 and began transitioning to becoming a Special District with six localities in 2023 [81–85]. This change might raise the interest of local advocates in accessibility assessments among constituencies concerned about the availability and quality of health services in their communities, as services will likely be concentrated in a few administrative districts.

### Radiotherapy services

In 2020, Cali registered five radiotherapy facilities in the Colombian Special Registry of Health Services Providers (REPS in Spanish) [86]. Radiotherapy services are located in four TAZ along the north-south corridor on the lower part of the western Andean slopes and foothills, which are well-off areas of the city; two services at Imbanaco Medical Center are in the same TAZ. Four services are on the city’s west and one, Fundación Valle de Lilí, in the south, all in well-off areas (Fig. 1).

**Fig. 2:**
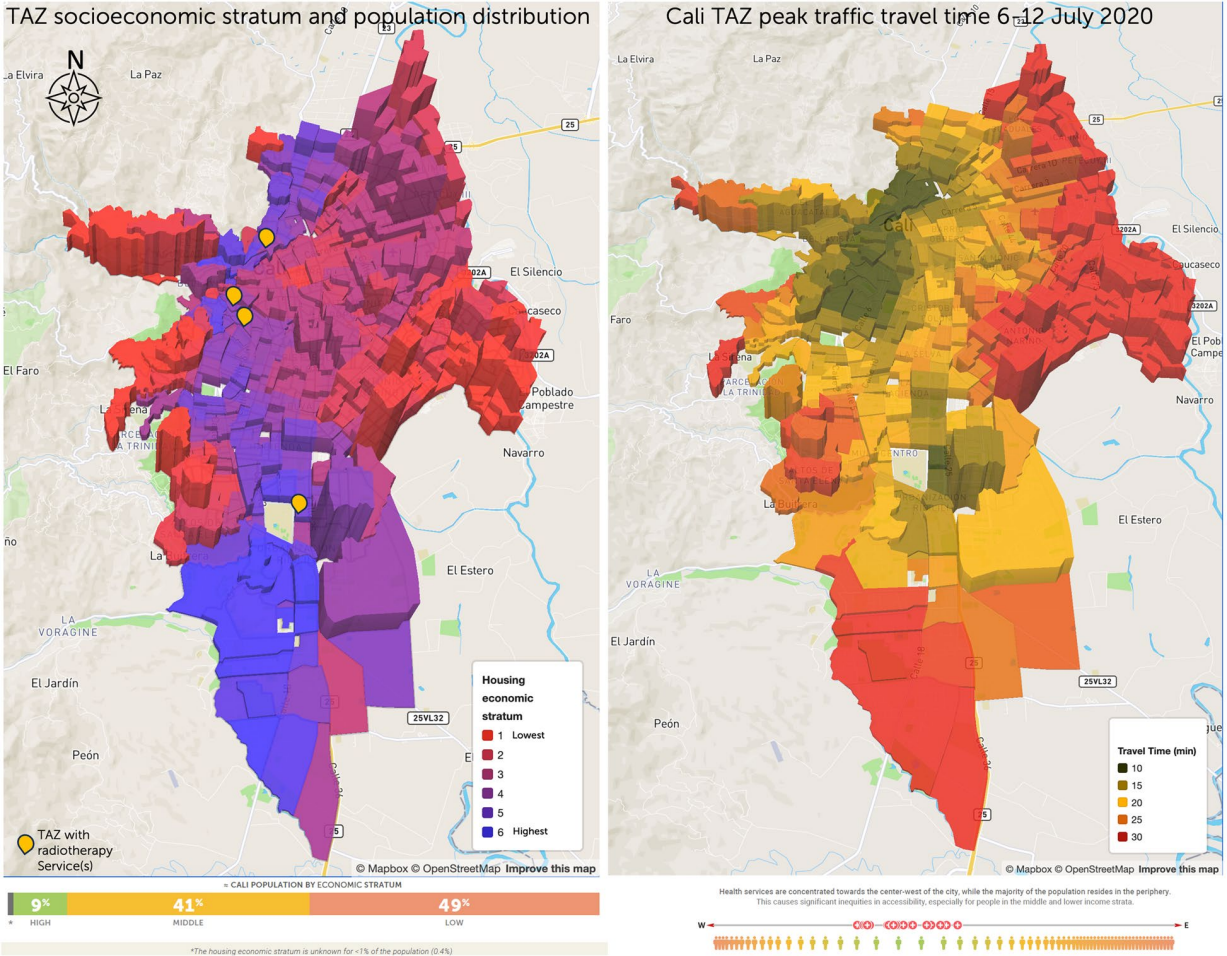
Population distribution of Cali’s 2020 TAZs by housing economic stratification and travel time

However, some high-income residents live in villas and mansions in southern Cali, and high-income housing hosts 9% of the population, including low-income resident homeworkers; almost half of the population lives in low-income areas, mainly in the city’s east and peripheric areas (Fig. 2) [80]. According to REPS, these radiotherapy facilities have extended hours from Monday to Saturday, providing crucial healthcare services.

Radiotherapy services are a sophisticated, expensive technology delivered by highly trained teams, with specialised radiotherapy modalities (e.g., 3D Conformal Radiotherapy, Intensity-Modulated Radiation Therapy, Image Guided Radiation Therapy, Stereotactic Body Radiation Therapy, Proton Therapy) beyond conventional radiotherapy (i.e., 2D Conformal Radiotherapy) There is an opportunity to look at the accessibility issues seeking to ensure a sound distribution of treatment modalities for existing service clusters and new centres to offer most common treatment options within reasonable travel times from where most of the population resides [57, 87, 88].

### Study design

This cross-sectional study used the AMORE web-based platform to integrate big data of travel times with georeferenced microdata from the adjusted 2018 national census, TAZs, and the geographic locations of radiotherapy services [12, 38, 89, 90]. The data sources integrated into the platform included the 2018 National Census Data for Cali, obtained from the public official databases of the Colombian National Department of Statistics (DANE in Spanish); [91, 92] administrative divisions of Cali obtained from the Colombian IDESC Geoportal, Traffic Analysis Zones (TAZs), and census block sectors [93, 94]; Google’s Distance Matrix API; and information about the five radiation therapy services in Cali. The study's complete list of variables can be found in the protocol and previous reports [12, 36, 38].

Travel times varied substantially during the COVID-19 pandemic. It is unclear how this influenced Google Distance Matrix algorithms [95]. As previously reported, everything indicates that these estimations remained accurate [36]. Radiotherapy facility data was obtained using REPS service codes 711 (radiotherapy as complementary or diagnostic treatment) and 408 (outpatient radiotherapy). Centres were registered under both service codes in 2020 and remained unchanged as of 30 November 2023 (Supplementary Material 5) [80, 86]. We did not include providers offering only nuclear medicine (code 715).

### Statistical analysis

Our report provides frequencies and bivariate analyses to represent cumulative opportunities as a measure of accessibility [79]. Critical variables for this report are travel time from the population weighed centroid of each residential TAZ to that of the TAZ hosting the radiotherapy service with the shortest journey time and the housing and sociodemographic characteristics of the population, as recorded in the Colombian national census microdata [38, 96]. The AMORE web-based platform displays population absolute and relative distribution (percentages) per travel time threshold measured for each of the nine traffic congestion levels, ranging from free-flow to peak traffic congestion [12]. We use isochrones or choropleths to represent the shortest median journey time required to reach a radiotherapy service at each hour of the day throughout the week. Univariate and bivariate analyses are described in the protocol and reports [12, 36, 38].

## Results / Outcomes

### Participants

**Fig. 3:**
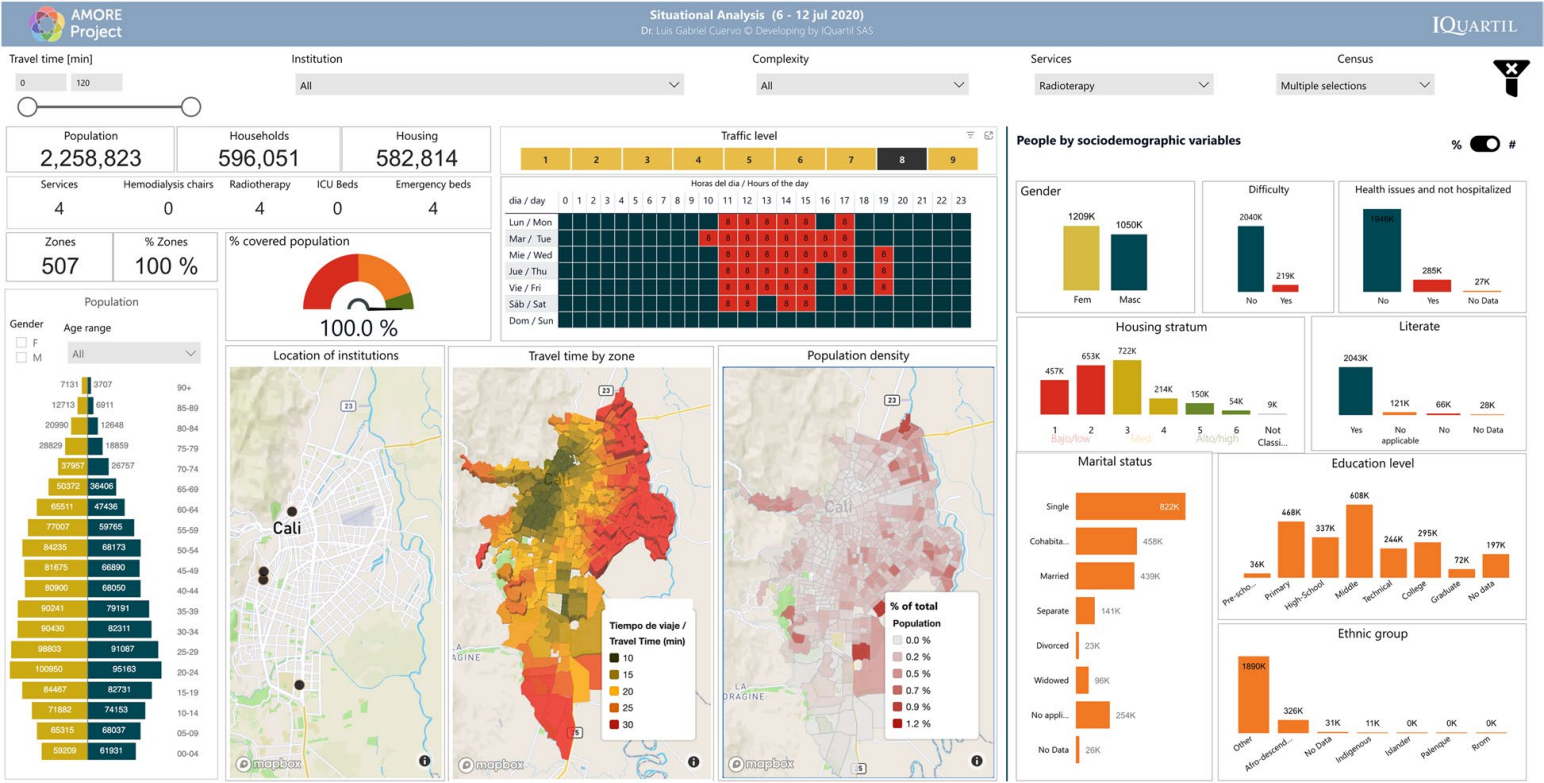
AMORE web-based Platform demographics interface (absolute figures) [98]

This study focused on the urban population of Cali registered in the 2018 census, adjusted to 2020 figures, with 2,258,823 residents [38]. Most of the population is mestizo or white (83.7 %, labelled in the census as “Others”) or Afro-descendants (326,492; 14.5%). Islanders (from the Department of San Andrés and Providencia) and Rrom people represent less than 1% of the population [97]. Residents are distributed in 507 TAZs, comprising 596,051 households within 582,814 housing units. Figure 3 illustrates how the AMORE platform’s interface displays the sociodemographic characteristics of the population, available from Tables 1 and 2, and previous reports describe the platform’s interface in detail [12, 36, 38].

**Table 1:**
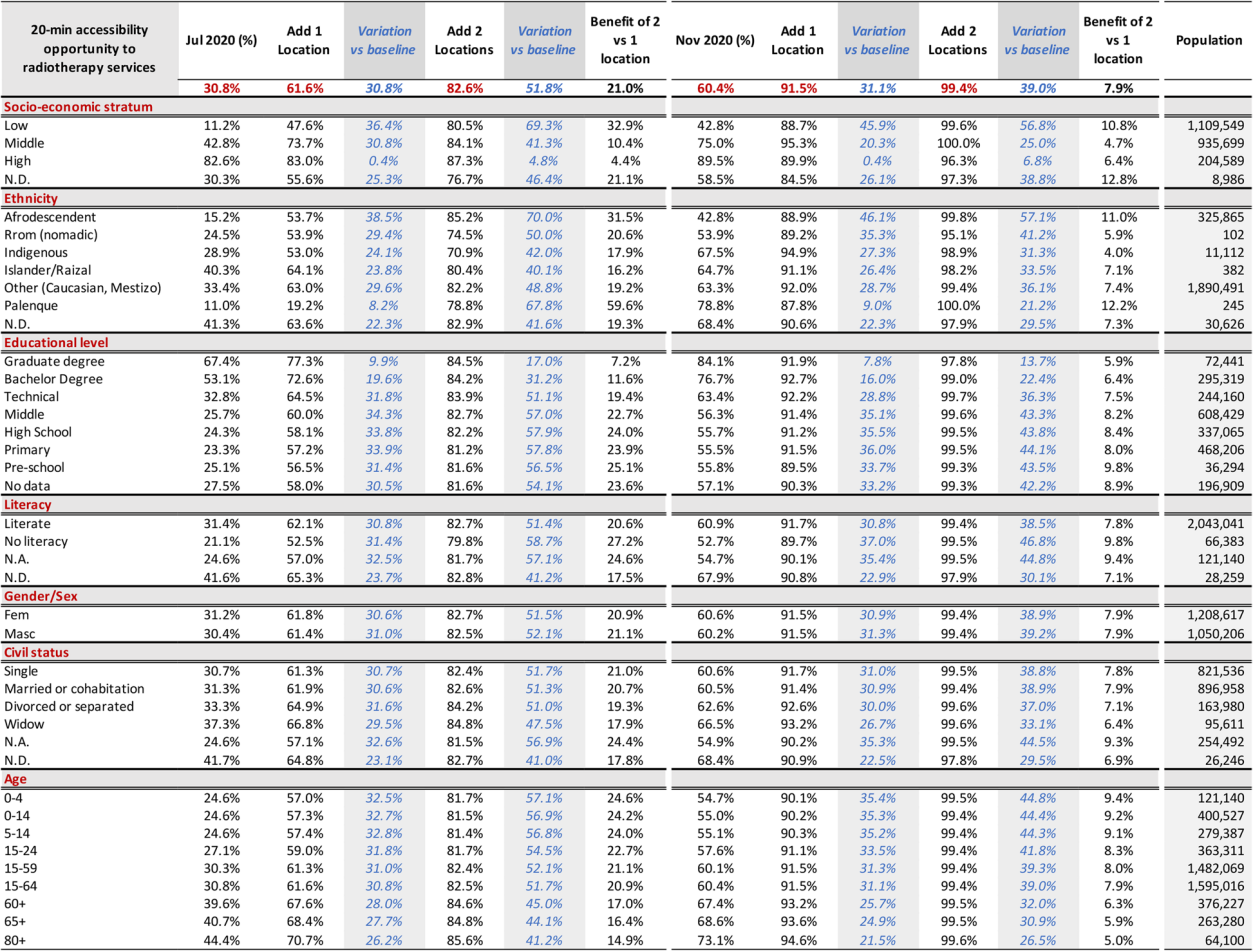
Relative figures: situational analysis and predicted 20-minute ACO by car to radiotherapy in Cali in July and November 2020

**Table 2:**
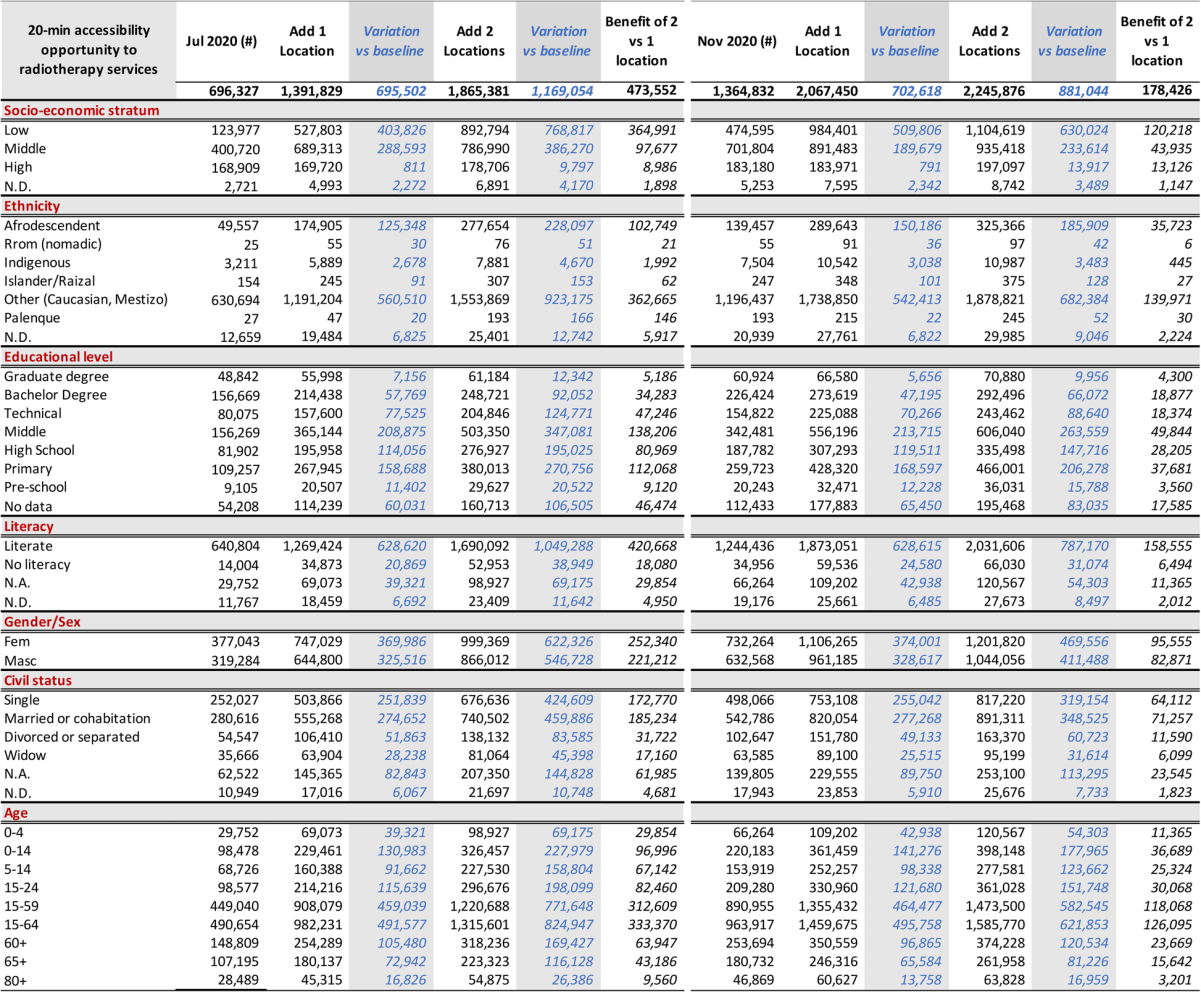
Population data: situational analysis and predicted 20-minute ACO by car to radiotherapy in Cali in July and November 2020

The socioeconomic distribution of dwellings for Cali shows that 1,109,549 residents (49% of the population) live in low-income housing. Most low-income housing is in the eastern and northeastern Cali and the western Andean slopes, including the areas along the road to the Pacific coast on its westerly limits. Most of the 935,699 middle-income Cali residents (about 41% of the population) live in 257,153 households in the central and southeast corridors of the city (Figs. 2 and 3) [12, 36].

Colombian national high-cost treatment monitoring services reported 527 new radiotherapy schemes (incident cases) in the five radiotherapy centres in Cali in 2020 (Cuenta de Alto Costo, Data request 5376, 29 May 2023. Supplementary Material 6). While most Cali residents, including those in vulnerable situations, live in densely populated outlying regions, radiotherapy services are in well-off areas with low population density (Fig. 2) [38].

### Baseline situational analyses

Figure 3 shows the situational analysis interface for Cali’s population in July 2020; Tables 1 and 2 present the peak traffic 20-min ACO and its distribution by housing income categorisation, and sociodemographic characteristics like ethnicity, gender, age, education level, and civil status per adjusted census [38, 98].

**Fig. 4:**
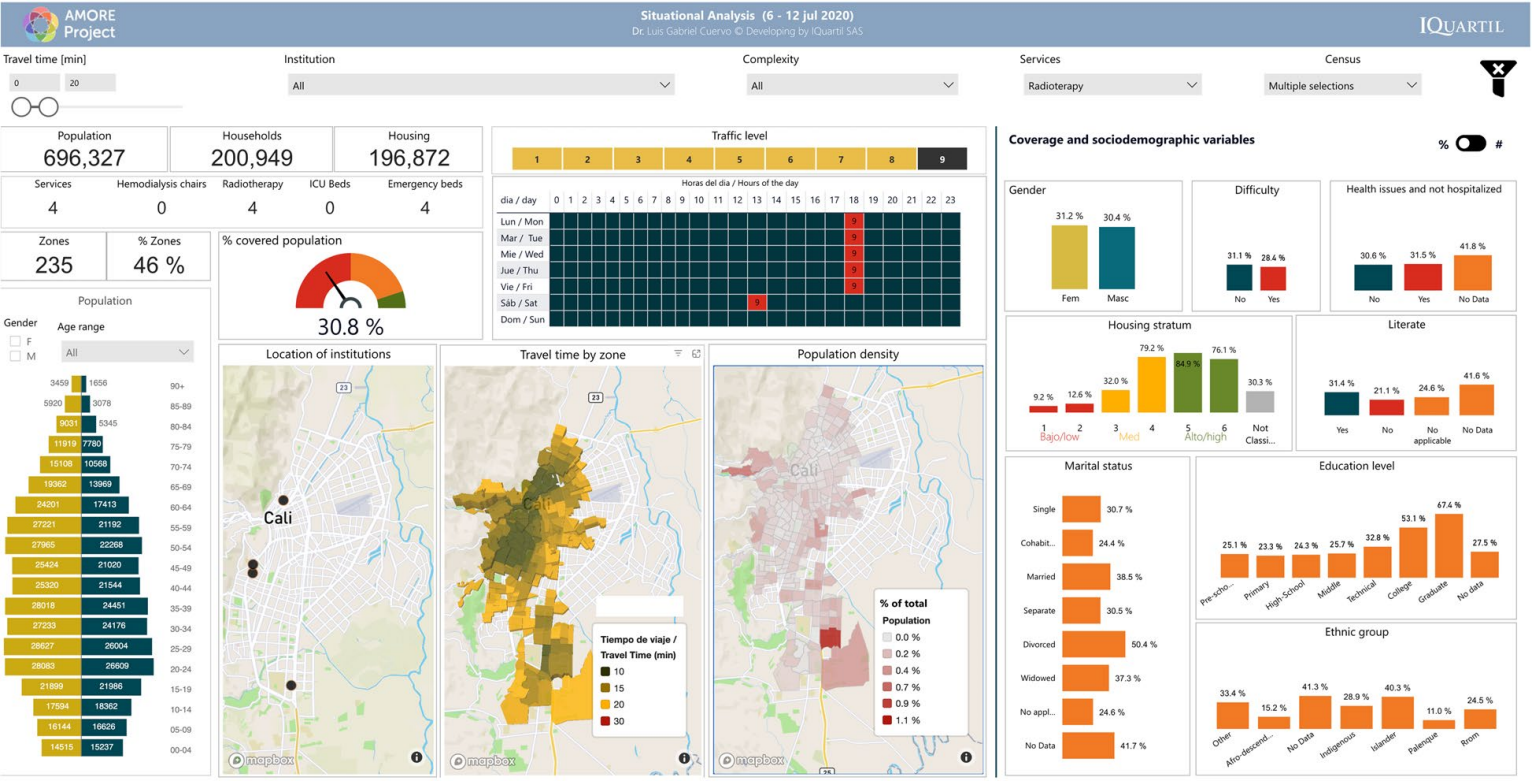
Relative 20-min peak-traffic ACO by car to radiotherapy, 6–12 July 2020 [98]

**Fig. 5:**
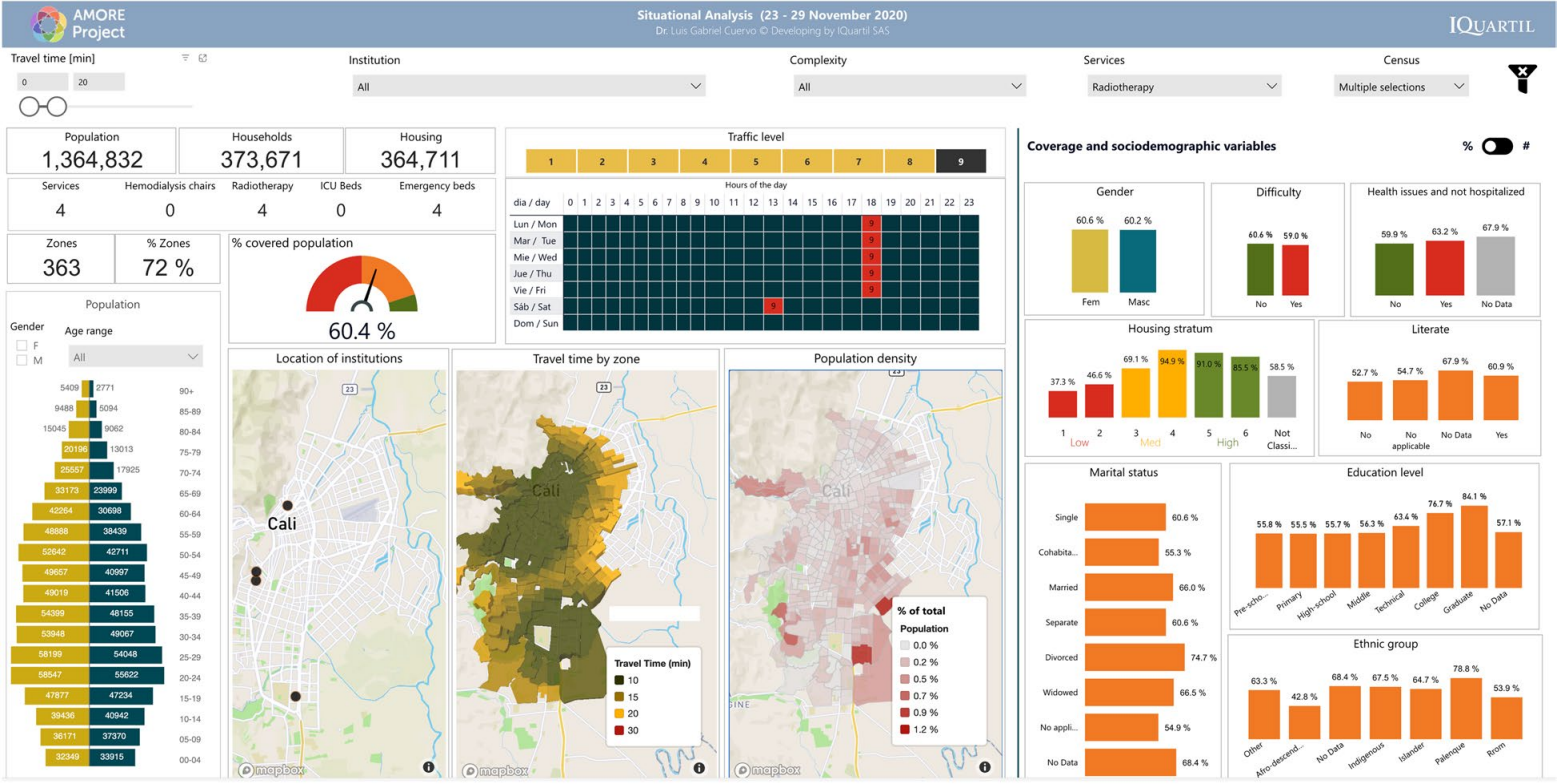
Relative 20-min peak-traffic ACO by car to radiotherapy 23–29, November 2020 [98]

**Fig. 6:**
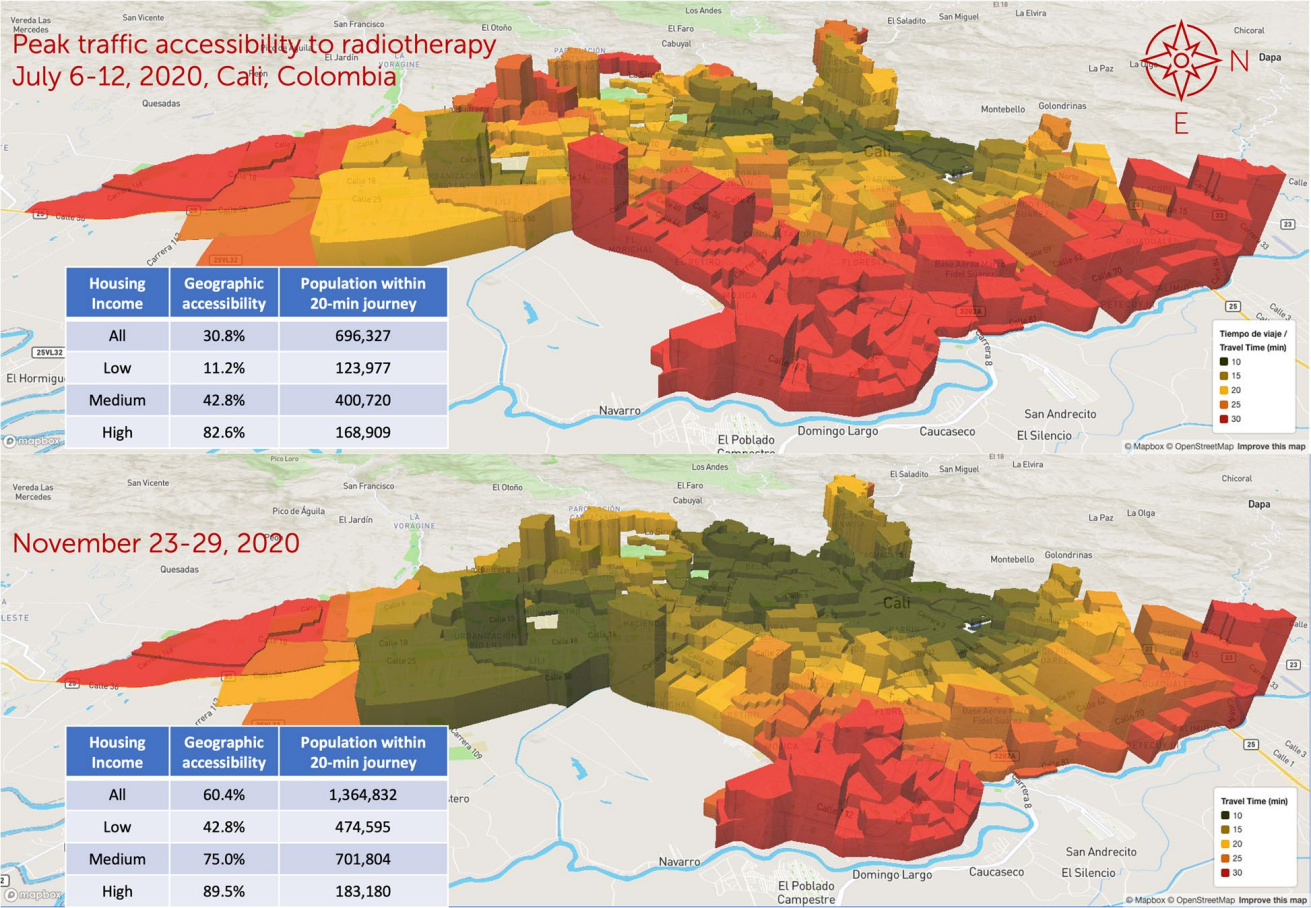
Assessed accessibility in July and November 2020 by economic stratum and location, with TAZ height representing population

Figures 4 and 5 show the interface for July and November, respectively, with a travel-time threshold of 20 minutes and relative population data [98]. Figure 6 complements these data, showing the territorial distribution variations for the July and November assessments and the matching population by housing income groups. It reflects improvements from reduced traffic congestion in November, when accessibility doubled.

### Effect of traffic variations (6–12 July vs 23–29 November 2020)

**Fig. 7:**
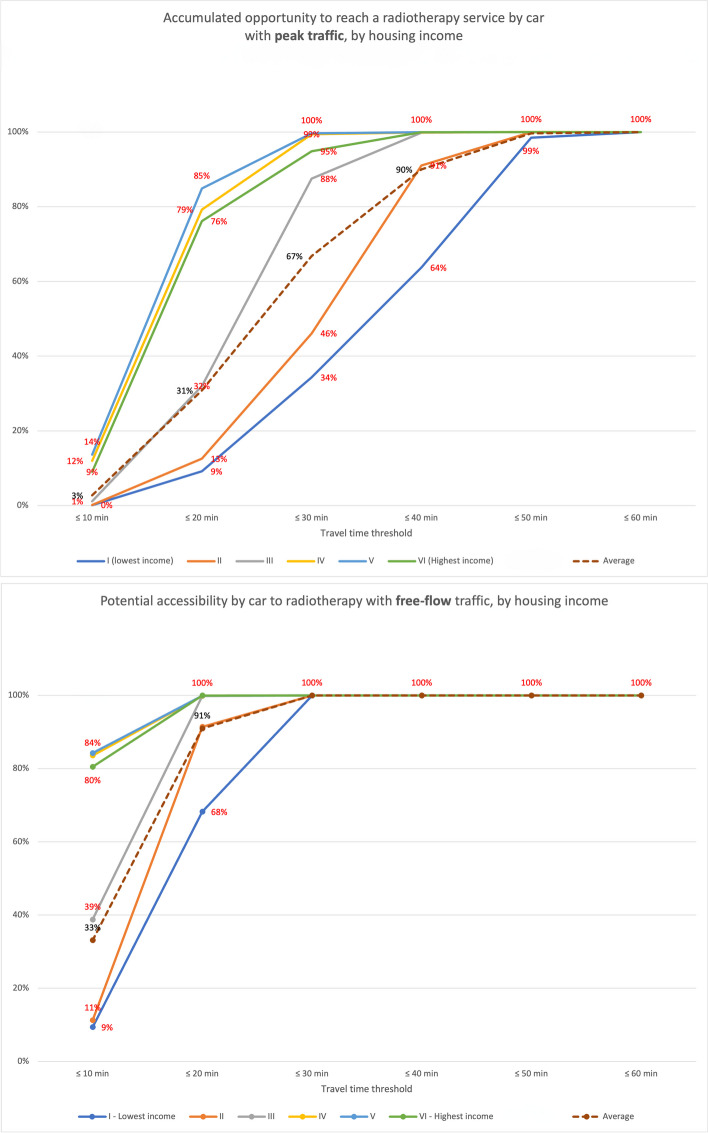
Accessibility gradients by housing economic stratum with peak vs free-flow traffic (6–12 July 2020)

**Fig. 8:**
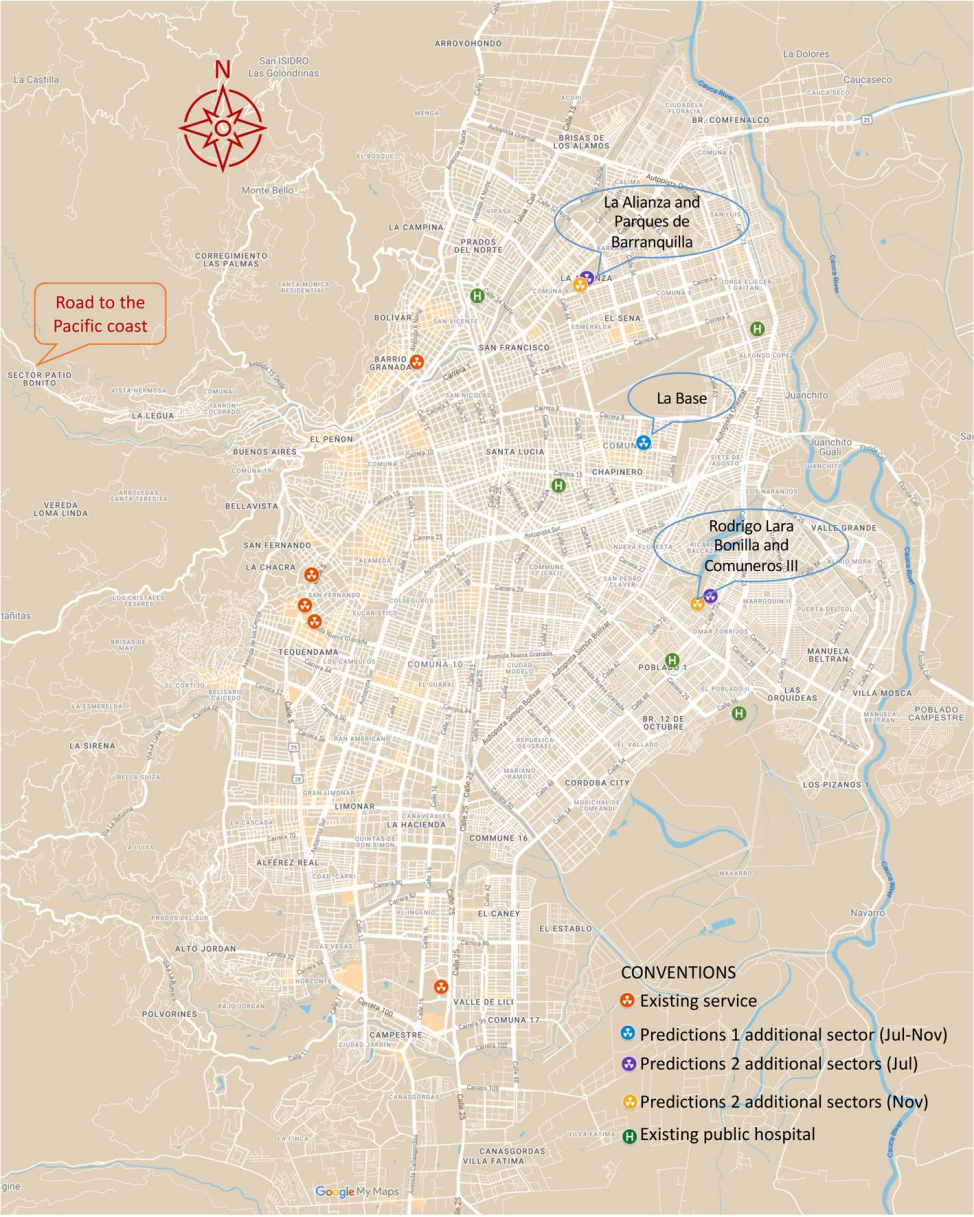
Locations to optimise urban accessibility by adding services in one vs. two sectors

**Fig. 9:**
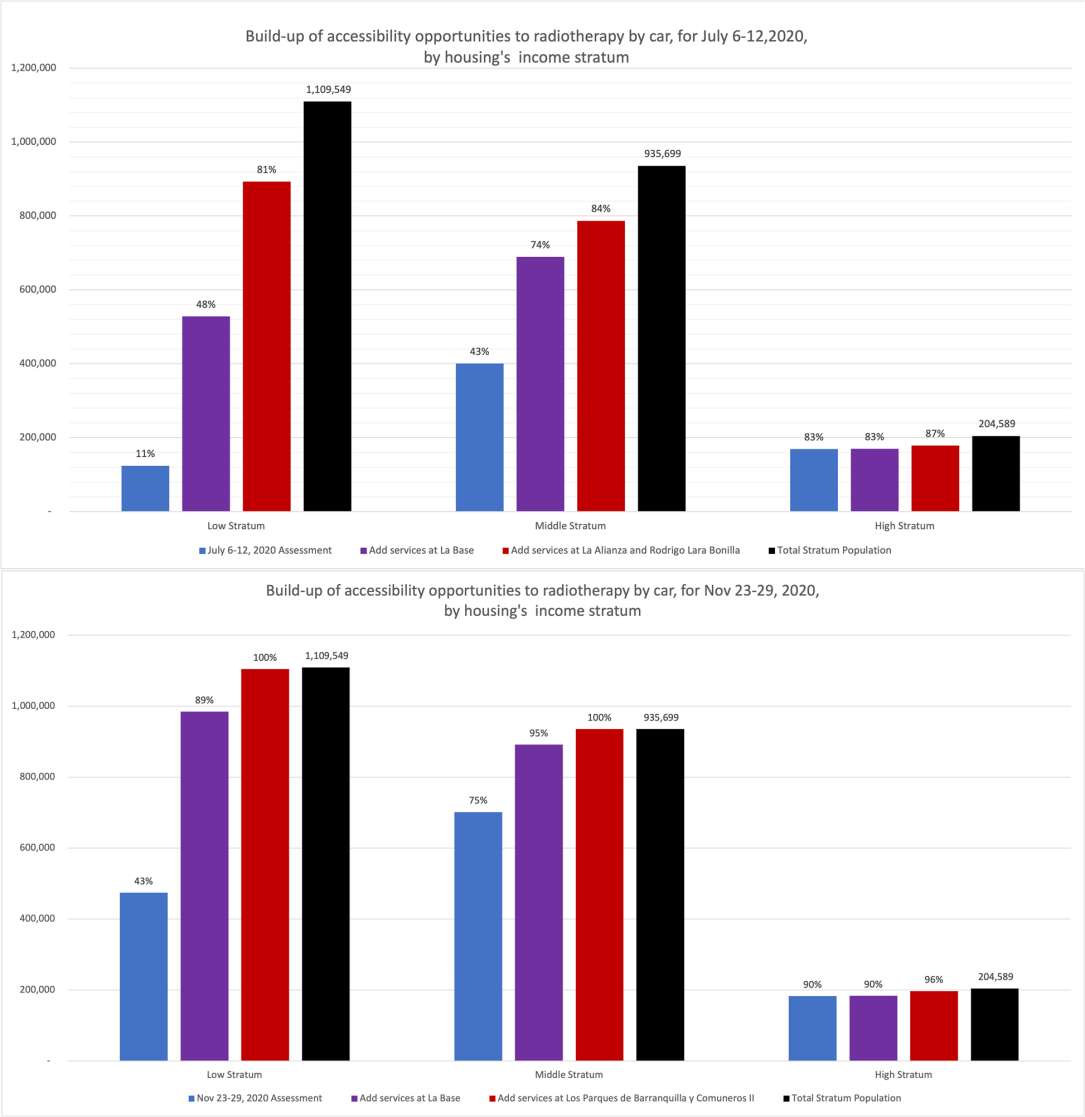
Assessment and predictions for accessibility improvement by housing income stratum for two measurement periods

We found that, due to heavy traffic congestion, most people living in low-income housing and the city’s periphery had lower geographic accessibility to radiotherapy services in November 2020 and July 2020 (Table 1, Figs. 4, 5, 6, 7, 8 and 9). Traffic congestion exacerbated inequities by disproportionately impacting the poorest populations in densely populated areas and the city’s outskirts (Table 1, Fig. 6, Supplementary Material 3 and Fig. 7); travel times are an access barrier to radiotherapy. Traffic congestion significantly decreased in the November measurements following the reinstatement of COVID-19 pandemic measures, such as stay-at-home orders and license-plate-based driving restrictions. In contrast, such measures were absent in July, leading to lower accessibility, particularly for residents in low-income housing (11.2% in July compared to 42.8% in November) and middle-income housing (42.8% in July compared to 75% in November). The change was less dramatic for those in high-income housing, with accessibility rates of 82.6% in July compared to 89.5% in November (Table 1, Figs. 6 and 9).

In July, Afro-descendants and the smaller Palenque community experienced the lowest accessibility rates, with 15.2% and 11%, respectively. In contrast, Islander and ‘Other’ populations (including whites and mestizos) fared significantly better, with accessibility rates of 40% and 33.4%, respectively. An earlier report showed that the small Palenque population is concentrated in a few neighbourhoods [12]. Because most of this population lives near the 20-minute ACO threshold borders, accessibility improved greatly when traffic congestion eased in November, reaching an accessibility rate of 78.8%, followed by indigenous communities at 67.5% (Table 2).

Individuals with graduate and bachelor’s degrees consistently experienced better accessibility and were less affected by reduced traffic congestion. Marital status also influenced accessibility, with married individuals or those in partnerships having lower accessibility in July (calculated to 31.3%, based on Table 2). However, as traffic congestion eased in November, accessibility rates became more uniform across different marital statuses, ranging from 60.5% for couples to 66.5% for widowers (Tables 1 and 2).

We observed an age-related gradient in accessibility, with older populations enjoying better access. Interestingly, all age groups benefited similarly from the traffic congestion relief (Tables 1 and 2).

### Effects of the addition of new radiotherapy providers

We analysed what peak-traffic accessibility would look like if new radiotherapy facilities were optimally located in 1–2 TAZs. Optimisation analyses for urban accessibility during peak traffic hours consistently pointed to adding new services in the eastern sector of *La Base* (Figs. 8 and 11). This finding was consistent for both measurement periods. Simulating the addition of this new service in July increased predicted urban accessibility from 30.8% to 61.6%. This addition expanded the 20-minute ACO to include 695,502 more inhabitants (Table 2).

This improvement remained substantial in November, with accessibility rising from 60.4% to 91.5% and incorporating 702,618 more inhabitants into the 20-minute ACO, markedly raising accessibility rates for all income levels and nearly all marital statuses, education levels, and ethnic groups. These results indicate that adding radiotherapy services in eastern Cali, specifically in or near La Base, will likely address critical health equity concerns related to accessibility (Tables 1 and 2 and Fig. 9).

Adding services in two new locations would notably increase Cali’s accessibility to radiotherapy services. While July and November predictions point to four different geographical areas, the recommended sectors are located nearby in the northeast and southeast (Fig. 9). July predictions showed that adding radiotherapy services in La Alianza in the northeast and Rodrigo Lara Bonilla in the southeast would increase peak-traffic ACO opportunities by 168%, reaching 82.6% of the population (Fig. 12), with 1,169,054 inhabitants incorporated into the 20-minute peak-traffic ACO (compared to the baseline). Similarly, November predictions revealed that locating services at Los Parques de Barranquilla in the northeast would accommodate around 450,000 inhabitants, and at Los Comuneros III in the southeast, it would serve about 842,000 into the 20-minute peak-traffic ACO (Fig. 8).

**Fig. 10:**
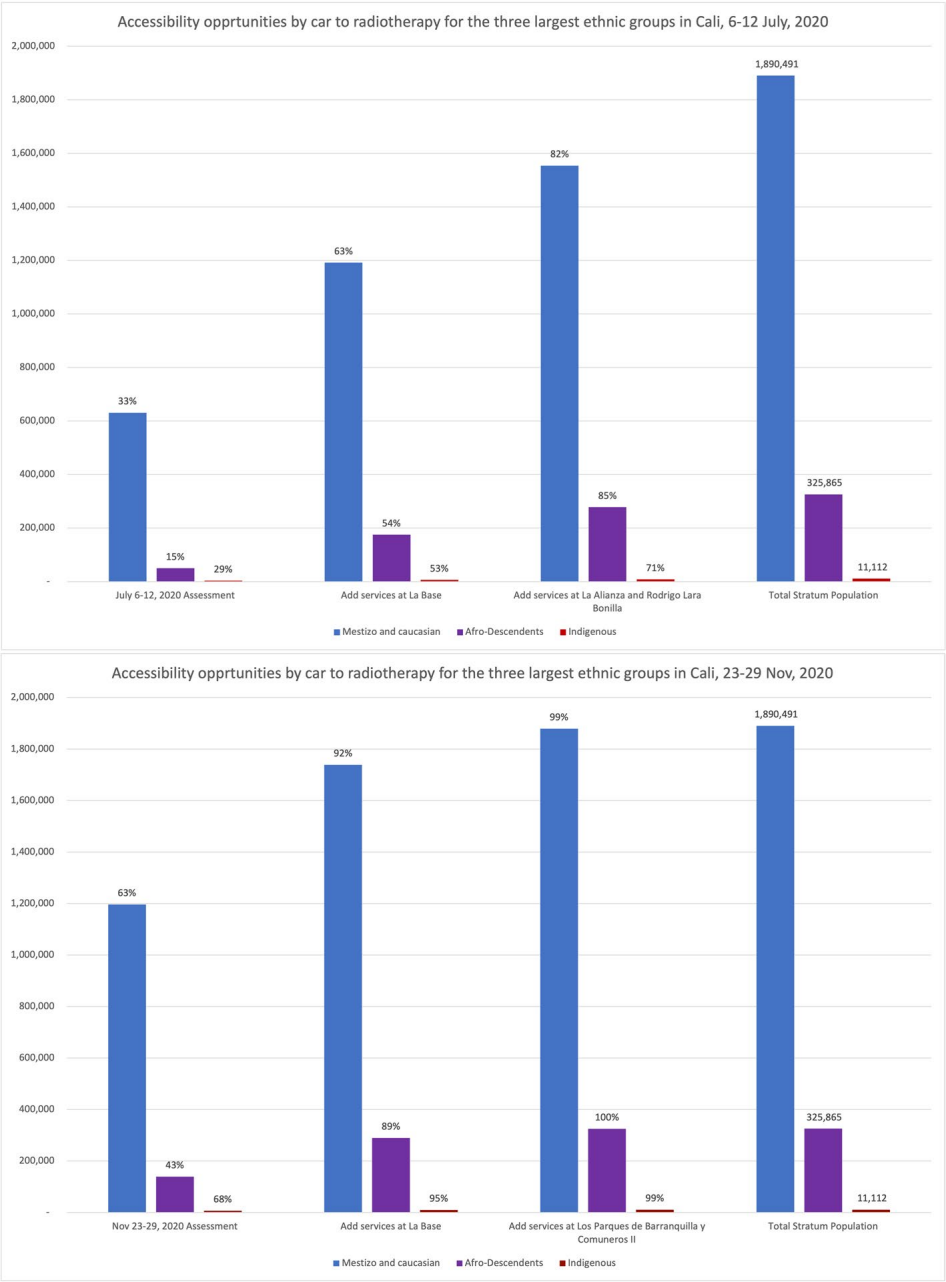
Assessment and predictions for accessibility improvement for the three largest ethnic groups in Cali during two measurement periods

**Fig. 11:**
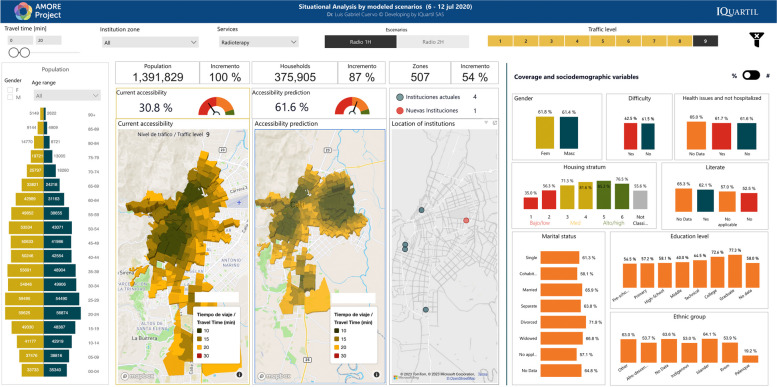
AMORE Platform interface showing the relative effects of adding services in one location based on July data

**Fig. 12:**
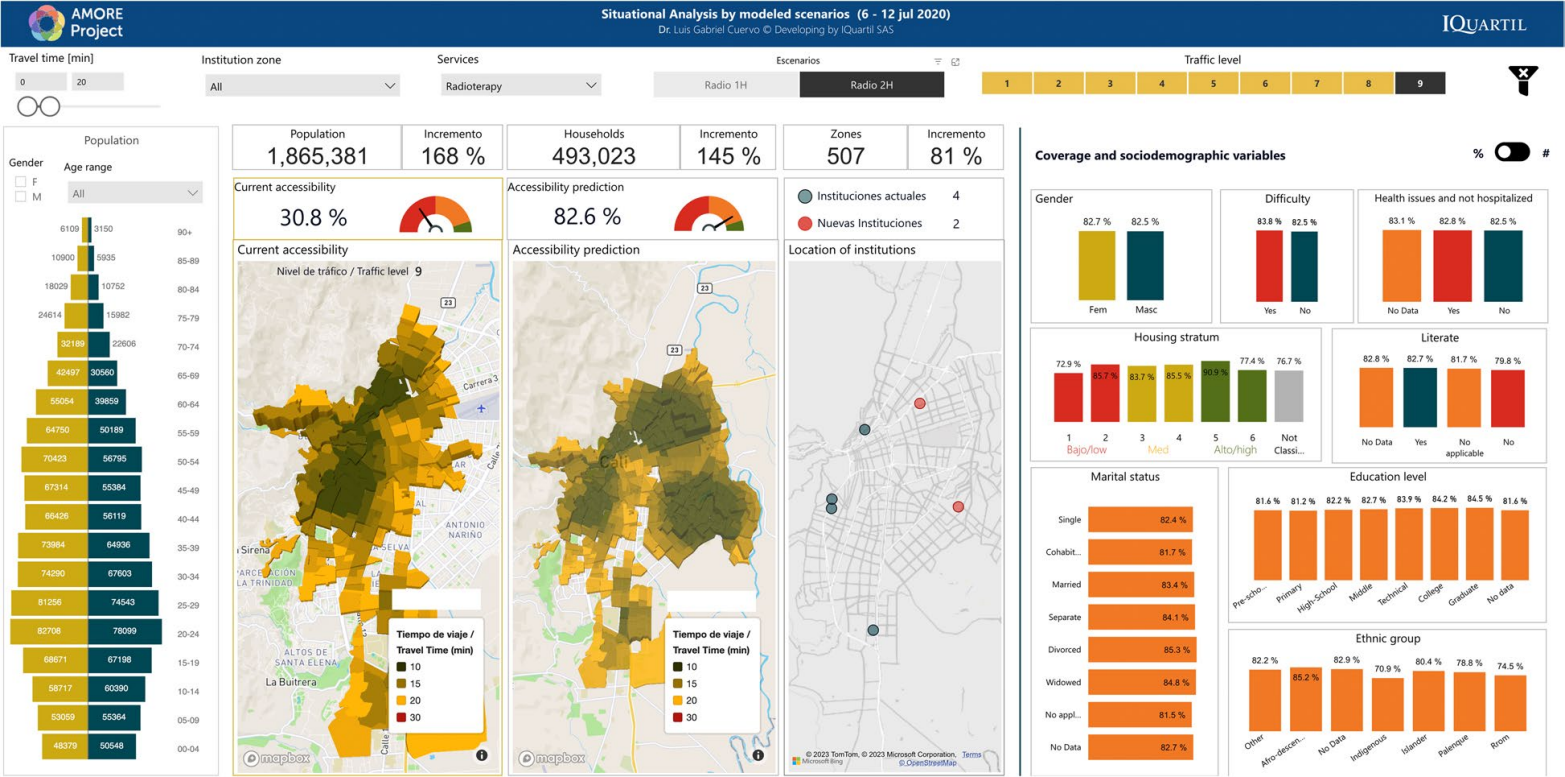
AMORE Platform interface showing the relative effects of adding services in two locations based on July data

The evidence, drawn from measurements in July and November, shows that introducing new radiotherapy services in recommended locations would improve accessibility, particularly in eastern Cali, and reduce inequities (Figs. 9, 10, 11 and 12). Moreover, the data also shows that it is better to add more sites. In July, the increase from adding two sites instead of one was 21.0%. In November, although lower by 7.9% (178,426), this increase still improves ACO for a large population (Supplementary Material 7 and 8).

### Other analyses

#### Comparing July and November 2020

We measured the gradients by which traffic fluctuations from free flow to peak traffic affect populations per housing economic stratum (Fig. 7). We found that the negative impact of traffic congestion is more remarkable for the lower-income stratum groups. We found a small high-income population living in villas in the city’s southern outskirts that remains beyond the 20-minute ACO.

## Discussion

The city’s layout and demography have changed since the city was concentrated around the corridor where services are located, and our findings offer a justification for updating land-use and health services plans. The new data provide insights for an equity-focused and data-driven approach that supports aligning the urban and health agenda [60, 99–101]. Our latest data indicates that populations in the peripheral areas and the densely populated city’s east must invest disproportionately when commuting to radiotherapy services. This should challenge traditional urban and planning approaches and support actions to enhance social justice by focusing on health equity [60, 71, 96].

Our findings highlight the potential for dramatic improvements by strategically locating radiotherapy services in 1–2 new sectors that could optimise accessibility. Traditionally, radiotherapy services have been co-located with tertiary care emergency services, but none exist in Cali’s east (Figs. 1 and 2) [12, 56]. Recently, stand-alone centres have been developed elsewhere, prompting consideration of whether Cali should have a tertiary care hospital(s) in the east offering comprehensive radiotherapy services or if stand-alone centres are more suitable.

If decision-makers add services in a single area, La Base or its surroundings emerge as the optimal location to maximise accessibility (Figs. 8 and 11). These services must have the capacity to meet the high demand from the populations covered in their catchment area and remain accessible to all radiotherapy patients. Expanding capacity in the east could alleviate demand pressure on existing centres, freeing up resources to accommodate the needs of metropolitan areas and neighbouring communities.

This study draws attention to the “inverse care law” in radiotherapy services in Cali, mirroring patterns seen in tertiary care emergency services and haemodialysis [12, 36]. Such a higher impact on vulnerable populations exacerbates social injustice [102, 103]. Previous studies have suggested that residents in informal settings, like others facing vulnerability and inequity in accessibility, are more likely to be disconnected from formal transportation modes. Video testimonies (close captioned with automated translation) from Cali’s peripheric impoverished areas reveal compounding mobility and financial challenges residents face trying to reach a paved area where private or hired drivers can pick them up to attend ambulatory services requiring frequent use, such as cancer treatments. They describe incurring catastrophic health expenditure and losing their autonomy. In addition to prolonged travel times, they may need help getting to where they can be transported to the service and still struggle to find suitable vehicles and the means to pay for their long commutes [3]. These testimonies broaden our understanding of the social justice issues that sink families into poverty and the consequences of keeping these populations beyond the reach of suitable public transportation [42, 48, 49]. The contrast is striking against the well-off locations hosting radiotherapy services and offering cheap and convenient commutes for neighbouring populations. This study underscores the imperative of bringing services within a short journey of patients.

Since radiotherapy is an integral part of a comprehensive treatment, service providers should avoid fragmenting healthcare services to maximise accessibility and quality of care [54, 55, 104, 105]. Recognising that radiotherapy capacities are strained, many individuals face long journeys to their service providers and that health services fragmentation makes things worse because they may not even be able to use the services with the shortest travel times, our findings are intended to encourage and inform societal agreements to improve accessibility to radiotherapy services [37, 53]. The challenges found in Cali likely extend to other cities in Colombia and beyond, amplifying inequities [90, 105]. The study’s approach has the potential to assess and monitor such issues in various contexts and enable participatory urban planning with inputs from advocates and civic groups [37, 42, 44, 104, 106–109].

This study’s approach opens avenues for implementing accessibility monitoring and evaluation, suggesting periodic (e.g., annual, semi-annual) assessments and exploring the economics of different approaches to enhance accessibility. For instance, by informing development plans, land-use and health services planning about adding services to existing public infrastructure, as tested in early AMORE web Platform prototypes, versus new infrastructure [77]. The expected release of projected census data in 2024 allows updating these findings and integrating this approach into urban observatory networks [110, 111]. This also presents a chance to promote community data initiatives involving intersectoral and multistakeholder collaboration. For instance, addressing the scarcity of data from informal settlements, popular neighbourhoods, or Cali’s new districts to tackle health equity and social justice issues for populations that often lack agency and remain neglected [41]. Another opportunity for future development involves applying the approach to specialised radiotherapy services, such as brachytherapy.

Our findings emphasise the need to monitor demand, building on open and big data integration opportunities [30]. Epidemiological and economic studies could shape the services and capacities of new and existing radiotherapy facilities. Understanding current and predicted radiotherapy needs and expected trends would benefit such deliberations.

Another practical application is calculating a fairer distribution of subsidies by adjusting them according to DSTAM. These measurements could be used to prioritise the organisation of mobility services for patients in outlying urban areas, and extended to cover metropolitan areas [63, 112].

Given the substantial expected demand that strategically located services could have, our findings underscore the urgent need for additional radiotherapy services accessible to all residents. Our data provides insights and valuable parameters for health services planning and advocating for re-evaluating Cali land-use policies.

Despite its limitations, the new methodological approach tested for tertiary care emergency services, haemodialysis, and radiotherapy reveals the potential for notable improvements to accessibility, social justice and health equity if services were added to Cali’s densely populated eastern Aguablanca district. In this district, optimal locations for new services would optimise accessibility [12, 36, 80]. Whether that is done through concerted action for specific services or an integral approach to improve multiple services at once (e.g., tertiary care emergency services, ambulatory treatments for high-cost conditions) is beyond the scope of this manuscript. The territorial distribution of existing services makes these findings unsurprising, and this study and the AMORE web-based interactive platform reveal the links between the population, the territory, and services, with cartography, measurements and predictions pertinent to public policy and urban planning.

### Key results

The impact on health equity, as our findings show, is substantial and multifaceted. Our findings predict that accessibility could be quickly improved by adding strategically located radiotherapy services and allowing patients to access services offering the shortest journeys. That could transform accessibility opportunities throughout the city, foment new data-driven approaches, and spur further development and innovation to improve the quality of care [51, 53, 60, 101]. We found that it is possible to enhance peak-traffic accessibility levels to levels currently seen only with free flow or minimal traffic congestion. Our predictions point to solutions worth considering. We have informed stakeholders at every level of primary healthcare governance, enabling them to advocate, plan, and monitor [13, 21, 35, 38, 75]. These stakeholders include government, communities, service providers and users, opinion leaders, local leaders, academics, patient groups, scientists, and the media [23, 25, 35, 37, 38, 59]. Those entities actively engaged with this participatory research project are of particular significance [3, 22, 35, 113]. We encourage policymakers to explore strategies for fairer transportation subsidies by grading them according to accessibility.

Our evaluations could be replicated, perfected, and further developed with multistakeholder groups supported by data scientists to generate repeated geographical accessibility assessments for these and other services in urban and metropolitan areas where traffic congestion matters [12, 14, 30, 36, 37, 51, 53, 77, 101]. We encourage adopters of this approach to use relatable formats where the data speaks for itself and where stakeholders can test assumptions and scenarios for themselves and find that they can communicate these findings to their constituencies and partners [13–15, 18, 21, 23, 25, 108, 113–115].

This study provides a valuable analysis of accessibility opportunities within the Colombian health system. It also sheds light on a practical estimation of traffic congestion’s impact on equity and accessibility. It is informative to stakeholders from diverse sectors (e.g., health, urban planning, smart city, economy) with a stake in urban and health services planning [27, 37, 53, 61, 100, 101].

### Interpretation

These findings empower citizens to participate in data-driven dialogues with land-use and health services planners. The overarching goal is to facilitate necessary improvements, bridging the health equity gap and ensuring universal access to healthcare [116].

Care models for bustling cities should incorporate quality aspects of health services like travel times. This analysis is an opportunity to recognise that comprehensive care for radiotherapy patients is inherently linked to other services, including psychological support, rehabilitation therapies, nutritional guidance, pain management, or palliative care. Therefore, to ensure that patients can access services without suffering hardship, planners must organise to offer integral care within reach, including chemotherapy and other ambulatory services that can be combined with radiotherapy [52, 53, 55–57, 87, 104]. Future research and smart governance could monitor and optimise the distribution of treatments with an integral perspective to minimise travel and service fragmentation and to provide continuity with established integral cancer treatment teams [55, 104]. A refined version of the AMORE Platform could be used to monitor progress [51, 60]. Local governments could adopt strategic approaches and enhanced indicators for accessibility in cities with heavy traffic [53, 60].

In this context, balancing the advantages and disadvantages of centralising versus decentralising radiotherapy services is essential. The geographical segregation of populations in Cali (Fig. 2) and the concentration of scarce services in areas with low population density disproportionately reduce service quality for those populations already hindered by a broad array of social determinants of health and result in geographic exclusion [90, 117].

Radiotherapy services are concentrated in the area from the north-to-south corridor of Cali to the west end of the valley where the city lies, leaving eastern Cali uncovered (Figs. 1 and 2). Restrictions that prevent patients from accessing services available in every sector could further reduce accessibility. For instance, the Fundación Valle de Lilí radiotherapy service, in the southernmost part of Cali, is the sole provider for the densely populated southern areas of the city. Suppose health maintenance organisations or insurers (EPS/EAPB, in Spanish) do not contract with providers close to patients or assign patients to distant services. In that case, patients won’t benefit from services in their proximity. We consider that the low-accessibility and inequity gradients stress the urgency of implementing reforms to address the fragmentation of health service delivery.

Strategically adding radiotherapy services will benefit the overall population across all economic strata and ethnic groups, with advantages for currently underserved groups (see Tables 1, 2, Figs. 8 and 10). Future studies could measure the direct and collateral economic impact of putting services within shorter travel times and distributing transportation subsidies with schemes that support social justice.

Colombian law requires the treatment of emergencies throughout the health system, reducing fragmentation. However, such a law needs to be put in place for ambulatory services for high-cost conditions, like radiotherapy; therefore, patients might be restricted from accessing conveniently placed IPSs [12, 118]. Colombia is undergoing a health reform, and assessments like this could provide insights for policy change, including free choice for radiotherapy providers.

We encourage readers to access the platform to test assumptions and scenarios. The interactive platform allows one to toggle or select specific radiotherapy providers. The platform can be accessed at https://www.iquartil.net/proyectoAMORE/ with additional guidance at https://padlet.com/Proyecto_AMORE_Project/products [21, 98].

This and previous assessments done as part of the AMORE Project Collaboration’s proof-of-concept show it is possible in LMICs to integrate open and big data to obtain dynamic geographical accessibility assessments for health services, providing an equity perspective and following recommendations to communicate findings to multiple stakeholders better [3, 12, 29, 36].

### Generalizability

The availability of travel-time big data allows for assessing various transportation modes. This approach applies to a broad spectrum of services, provided georeferenced data is available.

Notably, international policies and mandates are accelerating digitisation and datafication trends in many countries [119]. This study’s approach is transferable and can be replicated in other settings with traffic congestion, provided the essential data is available. Using population-weighed TAZ centroids instead of blocks reduces the overall costs associated with travel-time data while allowing for precise estimates [3, 36, 38, 89]. Like Colombia, other countries can harness available georeferenced open data of their population and services and integrate it with travel-time big data and TAZs to estimate ACOs [119, 120]. Travel-time big data is available from various providers. The resulting assessments could inform land-use and health services planning and contribute to aligning radiotherapy and other cancer treatments with universal health coverage [29, 121].

The public and private sectors can also adopt and refine this methodology by integrating information about population epidemiology, insurance providers, and payment agreements with health providers. These approaches are more complex and require investing in technology and participatory methods for stakeholder engagement. For example, integrating additional data might help identify the service with the shortest journey and availability for a specific appointment time. It might also guide insurers’ prioritisation of providers to expand their coverage or reduce fragmentation. When patients can access services in every sector offering them, accessibility will reach the levels we measured. As we reported in this study, the benefits of obtaining more precise and sophisticated assessments should be carefully weighed against the required resources, considering the high-level evaluation feasible with reduced funding and in a short time.

Our methodology can be adapted to assess other transportation modes, services, and geographical areas. Such replication might involve downloading travel times for additional transport modes, adjusting traffic congestion models, population centroids within traffic analysis zones (TAZs), and refining predictive models [38].

While this study included approaches for promoting sectoral advocacy, social adoption and application of knowledge in health services planning, such assessments go beyond the scope of this report. Sustainability for these models can be achieved if travel-time metrics and monitoring become standard and a requirement in urban, health, and mobility assessments [53, 60, 101] .

### Limitations

This study seeks to identify the radiotherapy service with the shortest travel time. However, patients in Colombia are assigned to a health services provider institution (IPS in Spanish) under contract with the health promotion entity (EPS, in Spanish) or Benefits Plan Administration Company (EAPB) responsible for providing services to them [118]. It may result in reported accessibility rates exceeding the accessibility individuals experience, which will be lower when EPS affiliation limits a patient’s access to the services offering the shortest travel times. We had no access to georeferenced data on EPS/EAPB coverage for populations; therefore, our findings show cumulative opportunities instead of actual accessibility. This fragmentation introduces a potential bias in our findings.

This report does not consider the characteristics of individual radiotherapy services, their capacity, or which ones offer specialised brachytherapy services, for example. It also does not integrate service agreements or georeferenced patient information. Therefore, our assessment errs on being optimistic; it reflects those populations that, even in the best-case scenario, would be beyond the assessed travel-time threshold. It points out that addressing these issues may involve actions on different fronts, from adding services to addressing health services fragmentation to enable access to those services with the shortest journeys and providing an integral approach to cancer treatment where radiotherapy services are located. Nevertheless, it is possible to adjust for specific services by activating or deactivating services considered in the accessibility assessments in the AMORE Platform. For faster response, the published AMORE platform is set to identify locations that optimise accessibility with peak traffic [98].

Radiotherapy services are provided to the broader metropolitan area and neighbouring cities; this study focused on urban Cali. While the approach's efficiency relies on using open and big data, the accuracy of the findings depends upon the accuracy of these publicly available data sources. Inaccuracies in sources may render some estimations or conclusions inaccurate. We did not include cost-effectiveness analyses or accurate demand and capacity estimates for new services.

## Conclusions

This study advocates for using DGAMs to monitor accessibility in cities where traffic congestion might be amplifying inequities and as conditions, infrastructure and populations change [122]. It also demonstrates that predictions suitable for integrating health with urban and territorial planning can be made using open data. The engagement of local stakeholders improved the platform and the pertinence of the analysis options and placed the focus on the needs of data end users and beneficiaries.

The study pragmatically approached the singularities of bustling urban settings with a holistic approach considering the stakeholders, tensions, and challenges of territorial and health services planning. It measures accessibility per traffic congestion gradients with an equity perspective. It offers a simple and robust approach, provides baseline assessments, and predicts the impact of interventions building on readily accessible open data and accurate travel time big data. The data and analyses used to reach the conclusions presented here are drawn from multiple research tools and methodologies, including cross-sectional epidemiological study, participatory research, and AI and data sciences. In using this approach, we have aimed to provide an integrative perspective built on a robust and diverse evidence base.

The potential of improving accessibility to radiotherapy services for many patients stresses the urgency of acting on these data. We recommend prioritising new integrated radiotherapy or cancer care services in Cali’s densely populated east, considering locations that maximise accessibility and health equity. Subsequent updates and evaluations will determine if adherence to integrated knowledge translation and social appropriation of knowledge principles lead to participatory urban and health services planning. They might also establish if services in eastern Cali will increase service referrals from the broader metropolitan area and neighbouring municipalities where radiotherapy is in short supply.

We sought the engagement and input of diverse stakeholders, including local government authorities and experts, health services planners, organised civil society groups such as academia and urban observatories, health service providers, health services user groups, and knowledge brokers [26, 27, 38]. The impact of such engagement differs from this report’s focus [38, 78].

Integrating equity assessments into urban and health services planning is crucial in aligning urban and health agendas, and inequality monitoring is key to addressing the social determinants for health and promoting social justice [60, 109]. This approach can be refined and adapted for cities to improve data-driven planning and develop suitable indicators for monitoring accessibility when heavy traffic congestion is prevalent [40, 51, 60, 99–101, 123]. This study points to solutions to address traffic congestion’s impact on social justice and health inequities. The new data can be used as part of an integrated strategic approach to advance SDGs (3, 9, 10, 11, and 17) in cities with heavy traffic and provide a baseline for Cali. The strategic approach used addresses calls made by United Nations agencies [37, 51, 99–101, 109].

### Other information

Health services planners could further research the effects of people-centred participatory approaches with multistakeholder engagement to seek stakeholder engagement in shaping urban and health services planning and social justice. The AMORE Project interactive interface is publicly available, and stakeholders can assess diverse scenarios and parameters [21, 24, 35, 38, 74, 124].

## Supplementary Information

Supplementary Material 1. Reuse permission for the Populab participatory video documenting patient testimonies from Cali residents regarding commuting to health services for haemodialysis and cancer treatments from Commune 18—link https://youtu.be/4fUgx7osKfw.

Supplementary Material 2. GIF animation illustrating radiotherapy accessibility under different traffic congestion levels.

Supplementary Material 3. STROBE Checklist for cross-sectional studies.

Supplementary Material 4. Radiotherapy services identified through REPS under codes 711 (Radiotherapy as complementary or diagnostic treatment) and 408 (Outpatient radiotherapy).

Supplementary Material 5. CAC report for radiotherapy services, Cali, 2020.

Supplementary Material 6. Improving Radiotherapy Accessibility—AMORE Project https://youtu.be/lQsGmbGWyZI

Supplementary Material 7. Impact of adding services illustrated in three side-to-side slides.

Supplementary Material 8. AMORE Project Collaboration contributors.

## Data Availability

Data sources are listed in the text, the protocol, and the project’s websites. https://www.iquartil.net/proyectoAMORE/ [98] https://padlet.com/Proyecto_AMORE_Project/products [114]. Datasets are available at reasonable request.
