# Correction: Improving accessibility to radiotherapy services in Cali, Colombia: cross-sectional equity analyses using open data and big data travel times from 2020

**DOI:** 10.1186/s12939-024-02347-5

**Published:** 2024-12-11

**Authors:** Luis Gabriel Cuervo, Carmen Juliana Villamizar, Daniel Cuervo, Pablo Zapata, Maria B. Ospina, Sara Marcela Valencia, Alfredo Polo, Angela Suarez, Maria O. Bula, J. Jaime Miranda, Gynna Millan, Diana Elizabeth Cuervo, Nancy J. Owens, Felipe Piquero, Janet Hatcher‑Roberts, Gabriel Dario Paredes, Maria Fernanda Navarro, Ingrid Liliana Minotta, Carmen Palta, Eliana Martinez-Herrera, Ciro Jaramillo, on behalf of the AMORE Project Collaboration

**Affiliations:** 1 Departamento de Pediatría, de Obstetricia y Ginecología y de Medicina Preventiva y Salud Pública. Facultad de Medicina - Edificio M, Universitat Autònoma de Barcelona, Campus Universitario UAB, 08193 Bellaterra, (Cerdanyola del Vallès), Cataluña Spain; 2 Academia Nacional de Medicina de Colombia, Cra. 7ª # 69‑11, 110231 Bogotá, Colombia; 3 Johns Hopkins Bloomberg School of Public Health, Wolfe Street Building, W1015, Baltimore, MD 21205 USA; 4 IQuartil SAS, Cra 13A # 107A-47, 110111 Bogotá, Colombia; 5 Department of Public Health Sciences, Faculty of Health Sciences, Queen’s University, Carruthers Hall 204, Kingston, ON K7L 3N6 Canada; 6 Universidad Nacional de Colombia, Ave Cra. 30 # 45-03, 111321 Bogotá, Colombia; 7 Facultad de Medicina, Universidad de Antioquia, Cra. 51D # 62-29, Medellín, Antioquia 050010 Colombia; 8 Technical Cooperation and Capacity Development, City Cancer Challenge Foundation, 9 Rue du Commerce, Geneva, 1204 Switzerland; 9 Independent Researcher, 110221 Bogotá, Colombia; 10 CRONICAS Center of Excellence in Chronic Diseases, Universidad Peruana Cayetano Heredia, Av. Armendáriz 445 - Miraflores, 15074 Lima, Peru; 11 Sydney School of Public Health, Faculty of Medicine and Health, University of Sydney, Camperdown, NSW 2006 Australia; 12 Universidad del Valle, Cali, Valle del Cauca 760032 Colombia; 13 Junta Nacional de Calificación de Invalidez [National Disability Board of Colombia], 110111 Bogotá, Colombia; 14 Independent Content and Communications Consultant, Fairfax, VA 22032 USA; 15 Patient Advocate and Author of an Autopathography, 110231 Bogotá, Colombia; 16 WHO Collaborating Centre for Knowledge Translation and Health Technology Assessment for Health Equity, Bruyère Research Institute, University of Ottawa, Ottawa, ON K1N 5C8 Canada; 17 Independent Consultant On Emergency Medicine and Humanitarian Response, 110111 Bogotá, Colombia; 18 Regional Director, City Cancer Challenge Foundation, 110111 Bogotá, Colombia; 19 ProPacífico, Calle 35 Norte #6A Bis - 100, 760046 Cali, Valle del Cauca Colombia; 20 National Faculty of Public Health, Universidad de Antioquia, Cl. 62 #52-59, La Candelaria, 050010 Medellín, Antioquia Colombia; 21 JHU-UPF Public Policy Center, Departament de Ciències Polítiques I Socials, Universitat Pompeu Fabra (UPF), Barcelona School of Management (UPF-BSM), Barcelona, Cataluña Spain; 22 School of Civil and Geomatic Engineering of the Universidad del Valle, Cali, Valle del Cauca 760032 Colombia

**Correction** **:** **Int J Equity Health 23, 161 (2024)**

**https://doi.org/10.1186/s12939-024-02211-6**

Following the publication of the original article [1], the author group requested the addition of a Video Abstract to the article.

The original article [1] has been updated.
