# Supplementary figures and images for: Improving accessibility to radiotherapy services in Cali, Colombia: cross-sectional equity analyses using open data and big data travel times from 2020
Source: Int J Equity Health. 2024 Aug 15;23:161. doi: 10.1186/s12939-024-02211-6 (PMC11325712; doi:10.1186/s12939-024-02211-6)

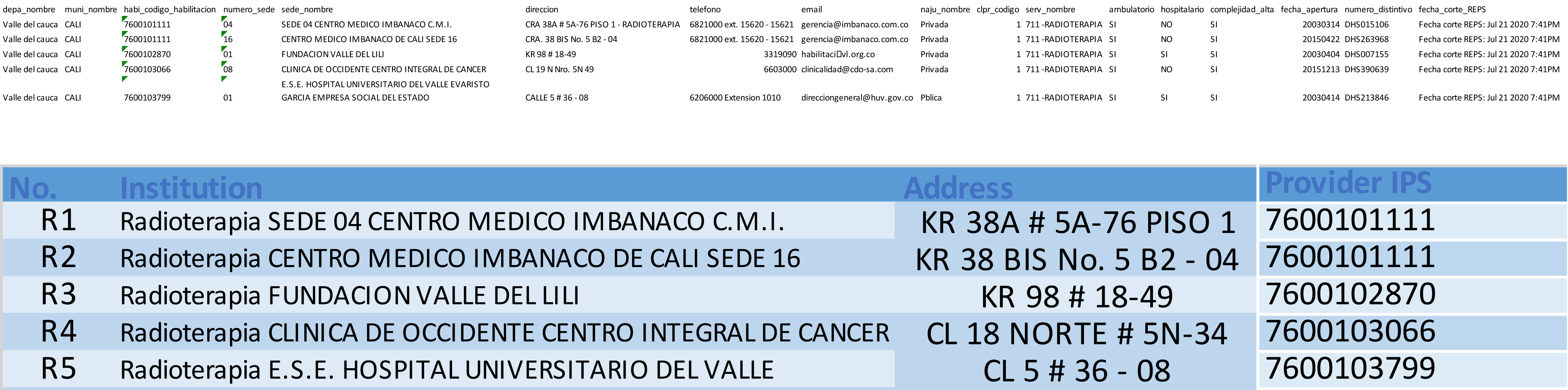

Supplement: Supplementary file 6 — Supplementary Material 4. Radiotherapy services identified through REPS under codes 711 (Radiotherapy as complementary or diagnostic treatment) and 408 (Outpatient radiotherapy). [file 12939_2024_2211_MOESM4_ESM.tif]

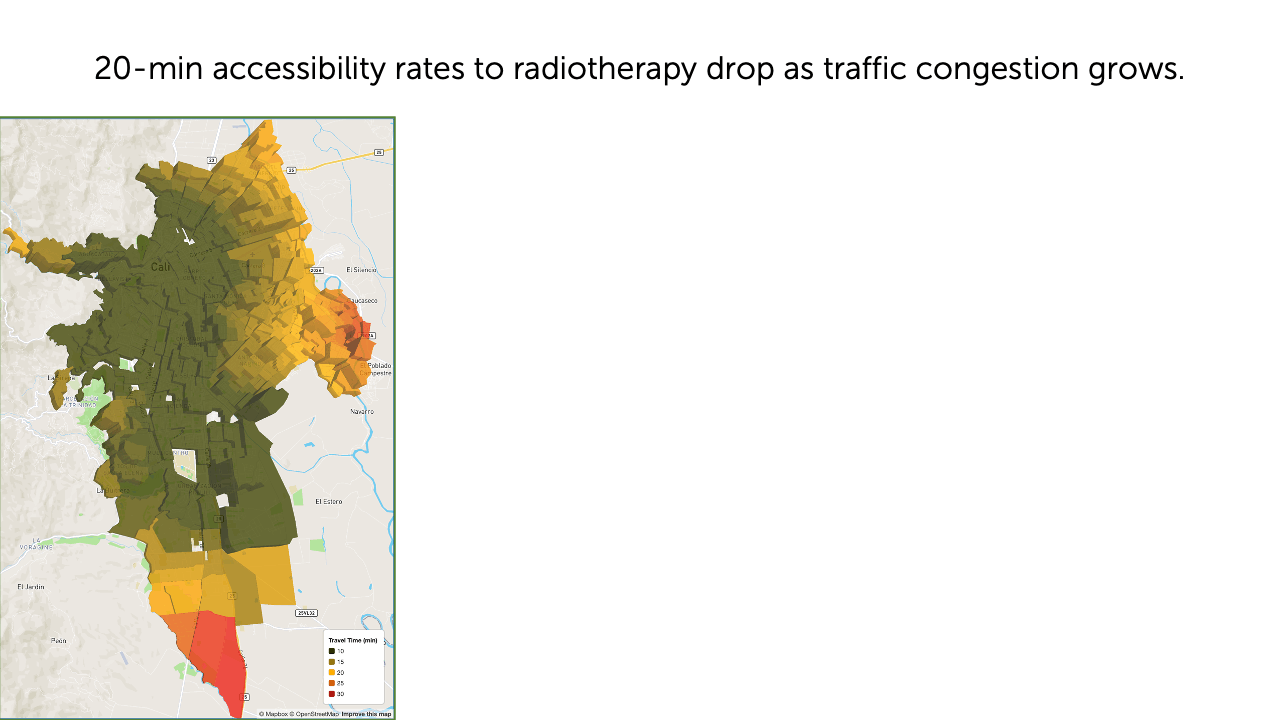

Supplement: Supplementary file 9 — Supplementary Material 7. Impact of adding services illustrated in three side-to-side slides. [file 12939_2024_2211_MOESM7_ESM.gif]

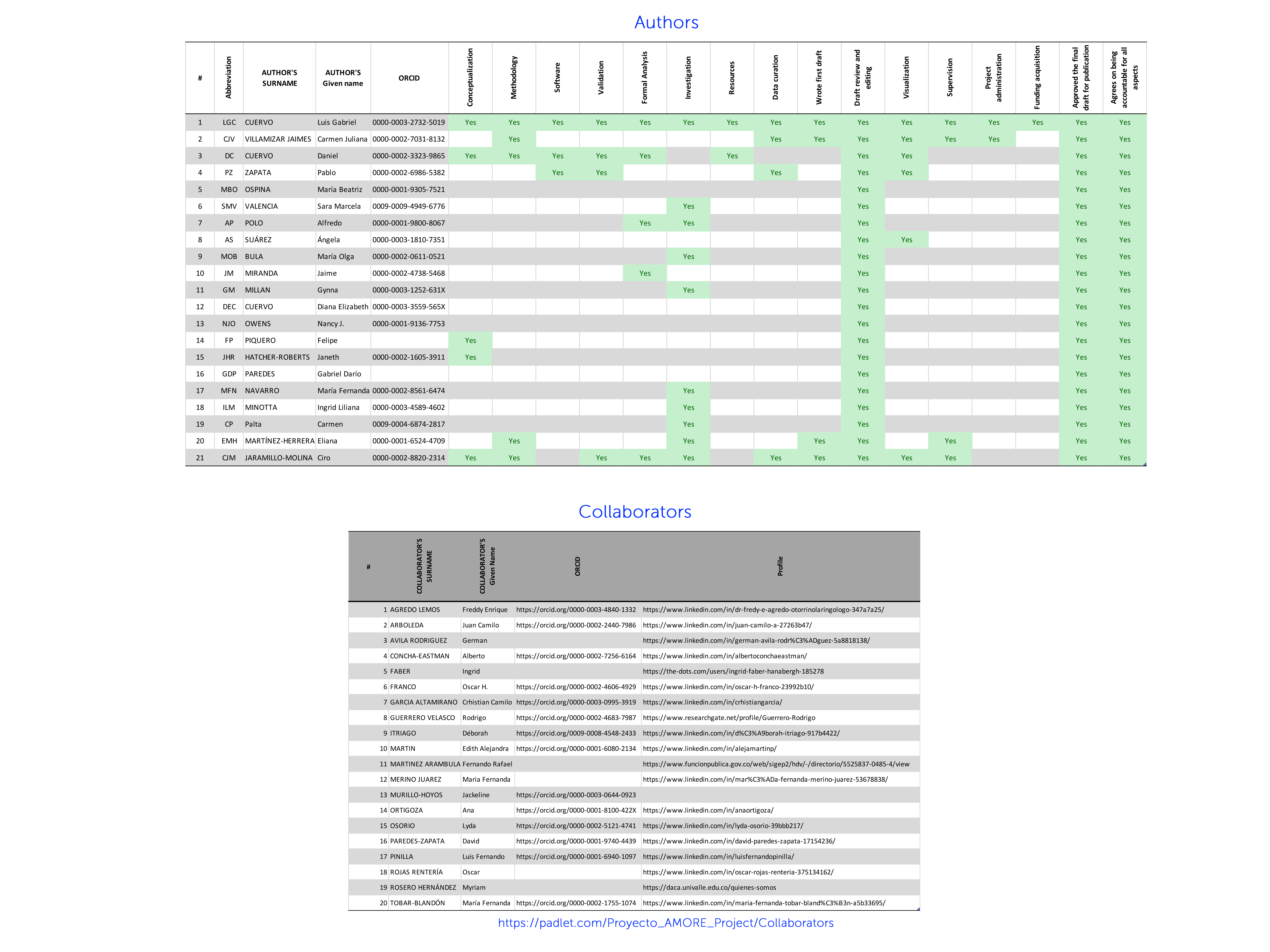

Supplement: Supplementary file 10 — Supplementary Material 8. AMORE Project Collaboration contributors. [file 12939_2024_2211_MOESM8_ESM.tif]
