# Supplementary material for: Improving accessibility to radiotherapy services in Cali, Colombia: cross-sectional equity analyses using open data and big data travel times from 2020
Source: Int J Equity Health. 2024 Aug 15;23:161. doi: 10.1186/s12939-024-02211-6 (PMC11325712; doi:10.1186/s12939-024-02211-6)
Supplement: Supplementary file 7 — Supplementary Material 5. CAC report for radiotherapy services, Cali, 2020. [file 12939_2024_2211_MOESM5_ESM.pdf]

Cuenta de Alto Costo
Solicitud de información CAC_5376

Estimación	Periodo	Tipo de cáncer	Casos que recibieron radioterapia (n)	Total de casos (n)	%
Proporción de casos incidentes que recibieron radioterapia	2021	Cérvix	41	111	36.94
Proporción de casos incidentes que recibieron radioterapia	2021	Colorrectal	42	242	17.36
Proporción de casos incidentes que recibieron radioterapia	2021	Estómago	26	183	14.21
Proporción de casos incidentes que recibieron radioterapia	2021	Linfoma Hodgkin	6	35	17.14
Proporción de casos incidentes que recibieron radioterapia	2021	Leucemia Linfoide Aguda	0	29	0.00
Proporción de casos incidentes que recibieron radioterapia	2021	Leucemia Mieloide Aguda	2	33	6.06
Proporción de casos incidentes que recibieron radioterapia	2021	Mama	122	551	22.14
Proporción de casos incidentes que recibieron radioterapia	2021	Melanoma	2	34	5.88
Proporción de casos incidentes que recibieron radioterapia	2021	Linfoma no Hodgkin	6	136	4.41
Proporción de casos incidentes que recibieron radioterapia	2021	Otros	195	1,668	11.69
Proporción de casos incidentes que recibieron radioterapia	2021	Próstata	59	258	22.87
Proporción de casos incidentes que recibieron radioterapia	2021	Pulmón	26	95	27.37
Proporción de casos incidentes que recibieron radioterapia	2021	Total	527	3,375	15.61

**Periodo**	**2021**	Corresponde al periodo de reporte comprendido entre el 02 de enero de 2020 al 01 de enero de 2021

Cuenta de Alto Costo
Solicitud de información CAC_5376

Estimación	Periodo	Tipo de cáncer	Número de casos con información	Promedio de sesiones	Desviación estándar
Promedio del número de sesiones de radioterapia por tipo de cáncer	2021	Cérvix	40	25.43	9.70
Promedio del número de sesiones de radioterapia por tipo de cáncer	2021	Colorrectal	41	21.49	11.77
Promedio del número de sesiones de radioterapia por tipo de cáncer	2021	Estómago	26	12.54	10.32
Promedio del número de sesiones de radioterapia por tipo de cáncer	2021	Linfoma Hodgkin	6	14.33	8.89
Promedio del número de sesiones de radioterapia por tipo de cáncer	2021	Leucemia Mieloide Aguda	2	2.00	0.00
Promedio del número de sesiones de radioterapia por tipo de cáncer	2021	Mama	121	8.28	5.07
Promedio del número de sesiones de radioterapia por tipo de cáncer	2021	Melanoma	2	17.50	17.68
Promedio del número de sesiones de radioterapia por tipo de cáncer	2021	Linfoma no Hodgkin	7	12.71	9.39
Promedio del número de sesiones de radioterapia por tipo de cáncer	2021	Otros	196	14.12	13.49
Promedio del número de sesiones de radioterapia por tipo de cáncer	2021	Próstata	59	19.44	13.95
Promedio del número de sesiones de radioterapia por tipo de cáncer	2021	Pulmón	25	8.84	7.44
Promedio del número de sesiones de radioterapia por tipo de cáncer	2021	Total	525	14.43	12.24

**Periodo**	**2021**	Corresponde al periodo de reporte comprendido entre el 02 de enero de 2020 al 01 de enero de 2021

Cuenta de Alto Costo
Solicitud de información CAC_5376

CUENTA DE ALTO COSTO
Fondo Colombiano de Enfermedades de Alto Costo

Intención del tratamiento del primer o único esquema de radioterapia (casos incidentes), Cali 2021

Tipo de Cáncer	Número de casos nuevos							
	Neoadyuvancia	Tratamiento Inicial curativo, sin cirugia sugerida	Adyuvancia	Manejo de recaída	Manejo de enfermedad metastásica	Manejo paliativo	Sin información	**Total**
Cérvix	8	10	7	15	0	0	1	41
Colorrectal	11	3	6	13	3	5	1	42
Estómago	3	0	9	2	7	5	0	26
Linfoma Hodgkin	0	1	2	2	1	0	0	6
Leucemia Mieloide Aguda	0	2	0	0	0	0	0	2
Mama	8	3	68	28	10	3	2	122
Melanoma	0	0	1	0	0	1	0	2
Linfoma no Hodgkin	1	2	0	0	3	0	0	6
Otros	23	15	102	28	23	3	1	195
Próstata	11	10	10	9	8	10	1	59
Pulmón	5	1	0	1	16	2	1	26
**Total**	70	47	205	98	71	29	7	527

**Periodo**	**2021**	Corresponde al periodo de reporte comprendido entre el 02 de enero de 2020 al 01 de enero de 2021	
